# Genome-wide identification and characterization of the *Populus* WRKY transcription factor family and analysis of their expression in response to biotic and abiotic stresses

**DOI:** 10.1093/jxb/eru381

**Published:** 2014-09-23

**Authors:** Yuanzhong Jiang, Yanjiao Duan, Jia Yin, Shenglong Ye, Jingru Zhu, Faqi Zhang, Wanxiang Lu, Di Fan, Keming Luo

**Affiliations:** ^1^Key Laboratory of Eco-environments of Three Gorges Reservoir Region, Ministry of Education, Chongqing Key Laboratory of Transgenic Plant and Safety Control, Institute of Resources Botany, School of Life Sciences, Southwest University, Chongqing 400715, China; ^2^Key Laboratory of Adaptation and Evolution of Plateau Biota, Northwest Institute of Plateau Biology, Chinese Academy of Sciences, 810008 Xining, China; ^3^College of Horticulture and Landscape Architecture, Southwest University, Chongqing, 400716, China

**Keywords:** Pathogen, *Populus*, SA (salicylic acid), stress tolerance, transcription factor, WRKY.

## Abstract

WRKY proteins are a large family of regulators involved in various developmental and physiological processes, especially in coping with diverse biotic and abiotic stresses. In this study, 100 putative *PtrWRKY* genes encoded the proteins contained in the complete WRKY domain in *Populus*. Phylogenetic analysis revealed that the members of this superfamily among poplar, *Arabidopsis*, and other species were divided into three groups with several subgroups based on the structures of the WRKY protein sequences. Various *cis*-acting elements related to stress and defence responses were found in the promoter regions of *PtrWRKY* genes by promoter analysis. High-throughput transcriptomic analyses identified that 61 of the *PtrWRKY* genes were induced by biotic and abiotic treatments, such as *Marssonina brunnea*, salicylic acid (SA), methyl jasmonate (MeJA), wounding, cold, and salinity. Among these *PtrWRKY* genes, transcripts of 46 selected genes were observed in different tissues, including roots, stems, and leaves. Quantitative RT-PCR analysis further confirmed the induced expression of 18 *PtrWRKY* genes by one or more stress treatments. The overexpression of an SA-inducible gene, *PtrWRKY89*, accelerated expression of PR protein genes and improved resistance to pathogens in transgenic poplar, suggesting that *PtrWRKY89* is a regulator of an SA-dependent defence-signalling pathway in poplar. Taken together, our results provided signiﬁcant information for improving the resistance and stress tolerance of woody plants.

## Highlight text

This study presents the genome-wide characterization of the *Populus* WRKY family under biotic and abiotic stresses. Overexpression of an SA-inducible gene, *PtrWRKY89*, enhanced resistance to pathogens in transgenic poplar.

## Introduction

Salicylic acid (SA) is a vital hormone and signal molecule in plants, synthesized from cinnamate in a reaction catalysed by phenylalanine ammonia lyase (PAL). The bulk of SA is produced from isochorismate in plants (Chen *et al.*, 2009*b*) and has many physiological functions such as temperature resistance (Dat *et al.*, 1998; Janda *et al.*, 1999; Clarke *et al.*, 2004), salt resistance (Borsani *et al.*, 2001; Tari *et al.*, 2002; Karlidag *et al.*, 2009; Palma *et al.*, 2009), drought resistance (Munné-Bosch and Peñuelas, 2003; Cho *et al.*, 2008) and ultraviolet radiation resistance (Yalpani *et al.*, 1994; Mahdavian *et al.*, 2008). Moreover, SA in plant defence responses is well-established because it is essential for the onset of the hypersensitive response (HR) and systemic acquired resistance (SAR) (Malamy *et al.*, 1990).

Induced-pathogen accumulation of SA, or treatment with SA, leads to a rapid increase in the levels of reactive oxygen species (ROS) via inhibition of the activity of SA-binding protein (SABP), which converts H_2_O_2_ to H_2_O and O_2_ (Chen *et al.*, 1993; Baker and Orlandi, 1995; Lamb and Dixon, 1997; Klessig *et al.*, 2000). The change in cellular redox potential results in the reduction of the NONEXPRESSOR OF PR1 (NPR1) oligomer to its active monomeric form, which is then translocated into the nucleus and enhances the binding of TGA transcription factors to SA-responsive promoter elements as a transcriptional co-activator of SA-responsive genes, such as pathogenesis-related gene (*PR1*) (Dong, 2004; Loake and Grant, 2007). In addition, NPR1 regulates the SA-mediated expression of *WRKY70* from *Arabidopsis*, which encodes an activator protein of SA-dependent defence marker genes (*PR1*, *PR2*, and *PR5*) (Uknes *et al.*, 1992). However, the induction of *WRKY70* expression by SA in *npr1* plants was only eliminated at later time points, implying that *WRKY70* expression is partially NPR1-independent (Li *et al.*, 2004). Recently, Shim *et al.* (2013) reported that AtMYB44 was an NPR1-independent regulatory component that directly regulated *WRKY70* expression. Therefore, SA-mediated defence signalling networks require transcription factors to regulate gene expression, which provides plants with a complex control mechanism against foreign pathogen attack.

In the past 15 years, major advances in WRKY transcription factor research have been made (Rushton *et al.*, 2010). The *WRKY* genes have been greatly expanded in plant genomes due to successive duplication events, resulting in large gene families that include up to 74 members in *Arabidopsis* (Ülker and Somssich 2004), 79 in *Arabidopsis lyrata* (http://supfam.mrc-lmb.cam.ac.uk/SUPERFAMILY), 197 in *Glycine max* (Schmutz *et al.*, 2010), 93 in *Sorghum bicolor,* 45 in barley (Mangelsen *et al.* 2008), and 81 in rice (Xie *et al.*, 2005). *WRKY* genes encode transcription factors characterized by the presence of one or two 60 amino-acid WRKY motifs, including a very highly conserved WRKYGQK sequence together with a zinc-finger-like motif CX_4–7_-CX_23–28_-HX_1–2_-(H/C) that provides binding properties to DNA (see Eulgem *et al.*, 2000, for a review). WRKY domains (WDs) probably evolved from an ancestral group IIc-like WRKY which derived from classical C_2_H_2_ ﬁngers (Znf) via an intermediate that was structurally close to the BED Zn-ﬁnger (Babu *et al.*, 2006; Brand *et al.*, 2013). The WRKY family members were classified into three groups on the basis of both the number of WDs and the features of their zinc-finger-like motif (Eulgem *et al.*, 2000). In general, members of group I typically have two WDs, whereas most proteins with one WD belong to group II. Members of group III contain a single WD, but the pattern of the zinc-finger motif is unique. Furthermore, group II has been divided into five subgroups according to phylogenetic analysis of the WDs (Eulgem *et al.*, 2000; Zhang and Wang, 2005). Most of the WRKY proteins bind to the conserved W-box TTGACY in the promoters of target genes related to the SA signalling pathway, such as chitinase (Yang *et al.*, 1999; Yamamoto *et al.*, 2004), and *PR* genes, such as *PR10* (Despres *et al.*, 1995; Rushton *et al.*, 1996). W-boxes have also been found in the promoter of *Arabidopsis* non-expressor of *PR* genes 1 (*NPR1*) as an important component of SA-mediated induction of *PR1* (Yu *et al.*, 2001).

More recently, increasing studies have focused on elucidating roles of WRKY factors in SA-biosynthesis, SA induction, and regulation of the signalling pathway. In *Arabidopsis*, the *wrky54wrky70* double mutant showed a remarkably high level of free SA compared to *wrky70* and *wrky54* single mutants, implying that *WRKY70* and *WRKY54* play a role as negative regulators of SA biosynthesis (Wang *et al.*, 2006). Bioinformatics analysis and expression studies demonstrated that WRKY28 and WRKY46 were transcriptional activators of *ICS1* and *PBS3*, which are involved in SA biosynthesis and SA-glucoside (SAG) accumulation, respectively (van Verk *et al.*, 2011). Yu *et al* (2001) categorized *WRKY3, WRKY4, WRKY6, WRKY7, WRKY15, WRKY21*, and *WRKY26* into an NPR1-independent group, and *WRKY62* to an NPR1-dependent group. *WRKY53* also showed normal expression at early stages but greatly decreased levels of transcripts at later times after SA treatment in the *npr1* mutant. Subsequently, it was reported that overexpression of *WRKY4* greatly enhanced plant susceptibility to bacterial pathogens and suppressed pathogen-induced *PR1* gene expression (Lai *et al.*, 2008). *WRKY38* and *WRKY62*, which were induced by SA in an NPR1-dependent manner, played a negative role in basal plant defence (Kim *et al.*, 2008). *WRKY18* was dependent on NPR1 in the plant defence response, and constitutive expression of *WRKY18* did not induce, but rather markedly potentiated, developmentally regulated *PR* gene expression (Chen and Chen, 2002). A few WRKY transcription factors have been functionally characterized in *Arabidopsis* and rice (Eulgem *et al.*, 2000; Rushton *et al.*, 2010), but our understanding of precise functions of WRKY members in the SA signalling pathway remains insufficient.

SA and jasmonic acid (JA) response pathways are one of the best studied examples of defence-related signal cross-talk in an antagonistic interaction (Pieterse *et al.*, 2009). For example, WRKY53 mediates negative crosstalk between pathogen resistance and senescence, which is most likely to be governed by the JA and SA equilibrium in *Arabidopsis* (Miao and Zentgraf, 2007). *WRKY70* is downstream of *NPR1* and *MYB44* in an SA-dependent signalling pathway. Plants overexpressing *WRKY70* showed constitutive expression of *PR* genes and suppression of several JA responses including expression of a subset of JA- and *Alternaria brassicicola*-responsive genes, indicating that WRKY70 has a pivotal role in determining the balance between SA-dependent and JA-dependent defence pathways (Li *et al.*, 2004; Li *et al.*, 2006; Shim *et al.*, 2013).

With the completion of the poplar genome sequence (Tuskan *et al.*, 2006), a number of WRKY members have been found in the whole-genome sequence data from *P. trichocarpa* Torr. & A. Gray (http://genome.jgi-psf-org/Poptrl_l/Poptrl_l.home.html). To date, however, there are only limited elaborate studies on the functional characterization of several *WRKY* genes in *Populus*. A recent study reported the molecular cloning and functional characterization of *PtWRKY23* in poplar (*P. tremula* × *P. alba*), which was induced rapidly by *Melampsora* infection and SA treatments, and expression patterns of *PtWRKY23* and *AtWRKY23* orthologues are not necessarily identical (Levée *et al.*, 2009), indicating that *WRKY* in poplar has a different function from *WRKY* orthologues in *Arabidopsis*. Misexpression of *PtWRKY23* in transgenic poplar plants led to increased susceptibility to *Melampsora* infection compared with the wild type, implying that this may be caused by deregulation of genes that disrupt redox homeostasis and cell wall metabolism. However, the role of *PtWRKY23* in the SA signalling pathway is still unknown.

In this study, we found that there is an expanded WRKY family with a total of 100 members in the *Populus* genome. A phylogenetic tree combining WRKY proteins from poplar, *Arabidopsis*, and other species was constructed to test their evolutionary relationships. Promoter analysis revealed that various *cis*-acting elements involved in stress and phytohormone responses were present in the promoter region of *PtrWRKY* genes. Transcriptome analysis showed that the majority (61) of the *PtrWRKY* genes were induced by the fungus *Marssonina brunnea* f.sp. *multigermtubi*, SA, methyl jasmonate (MeJA), wounding, and cold and salinity stresses. Furthermore, stress-response expression proﬁles were generated to screen candidate genes involved in signal transduction pathways initiated by SA in *Populus*. The function of an SA-inducible gene, *PtrWRKY89*, was characterized in transgenic poplar. Our results will be helpful for understanding the roles of the *WRKY* genes in poplar defence responses and provides valuable information for further identification of the functions of this signiﬁcant gene family in *Populus*.

## Materials and methods

### Plant growth conditions and treatments

#### Plant material and growth conditions 

*Populus trichocarpa* Torr. & A. Gray and *P. tomentosa* Carr. (clone 741) (Chinese white poplar) were grown in a greenhouse at 25°C under a 14/10h light/dark cycle.

#### Hormone treatments 

SA (5mM in water) and MeJA [1mM in 0.1% (v/v) ethanol] were applied at the different concentrations as 5ml droplets on each plant. The treated plants were immediately covered with a transparent lid. The leaves were collected after 24h (Li *et al.*, 2004). Additionally, the leaves applied for all stress treatments, pathogen infection, and RT-PCR analysis were excised from the second and third internodes.

#### Fungal inoculation 

Leaves of three-month-old plants were inoculated with *M. brunnea* f.sp. *multigermtubi* and *Dothiorella gregaria* Sacc., respectively. Mycelial plugs (6mm) were placed on excised leaves (at least three leaves for each plant). These leaves were incubated in Petri dishes with humid filter paper in a humid chamber for 3 d (Huang *et al.*, 2012).

#### Low temperature stress 

The healthy, well-hydrated plants were transferred to a growth chamber at 4°C under the same light and photoperiodic conditions for 1h. After cold treatment, plants were allowed to recover at 20°C for 1h.

#### Wounding stress 

For the wounding treatment, the young leaves of poplar plants were harvested after being punctured with sterile needles and placed at 20°C for 2h.

#### Salinity stress 

The four-week-old seedlings were subjected to salt stress. Saline treatments had the NaCl concentrations of 100mM added to full-strength Hoagland’s solution for 2 d. The method was described previously (Yang *et al.*, 2009*a*).

### Database search and sequence retrieval

The gene model IDs of the *P. trichocarpa* WRKY family were obtained from the DATF website (http://planttfdb.cbi.pku.edu.cn/index.php). The nucleotide and amino acid sequences of the *WRKY* genes were downloaded from Phytozome v9.1 website (http://www.phytozome.net/poplar). The amino acid sequences of these genes, which could not be detected in Phytozome v9.1 via ID numbers, were used as queries to perform BLAST searches against the *P. trichocarpa* genome database in Phytozome v9.1. After the identification of the *Populus WRKY* (*PtrWRKY*) genes, gene orthologue analysis was performed using the *WRKY* gene sets from *Arabidopsis* based on previously reported results (He *et al.*, 2012). The catalogue of WRKY proteins in poplar is shown in Supplementary Table S1. Sequences of the *AtWRKY* genes were searched and downloaded from the *Arabidopsis* genome TAIR 9.0 website (http://www.Arabidopsis.org/index.jsp). Sequences of WRKY proteins from other species were downloaded from NCBI (http://www.ncbi.nlm.nih.gov/) and the accession numbers are shown in Supplementary Table S2.

### Phylogenetic analysis

The alignments of the amino acid sequences of full PtrWRKY proteins and WDs were performed using Clustal X 1.81 (http://www.clustal.org/) (Thompson *et al.*, 1994, 1997). The parameters of alignment used were as follows: gap opening penalty, 10.00 (both in pairwise alignment and multiple alignment); gap extension penalty, 0.20 (both in pairwise alignment and multiple alignment); protein weight matrix, gonnet; residue-specific penalties, on; hydrophilic penalties, on; gap separation distance, 0; end-gap separation, on; use negative matrix, off; and delay divergent cutoff (%), 30. Phylogenetic trees were constructed through the neighbour-joining (NJ) method using program MEGA4.1 (http://www.megasoftware.net/mega.html) (Tamura *et al.*, 2007). The parameters of the constructed trees were: phylogeny test and options: bootstrap (1000 replicates; random seed = 9928), gaps/missing data: complete deletion, model: amino: Poisson correction, substitutions to include: all, pattern among lineages: same (homogeneous), and rates among sites: uniform rates.

### *In silico* analysis of regulatory elements in promoter region of *PtrWRKY* genes

The transcription start site was designated +1. The elements in the promoter fragments (from –1500 to +1bp) of *PtrWRKY* genes were found using the program PlantCARE online (http://bioinformatics.psb.ugent.be/webtools/plantcare/html/).

### Gene expression analysis

#### Digital transcriptomics analysis 

Digital gene expression (DGE) experiments were performed as described by Zhang *et al.* (2010). Briefly, total RNA was isolated from poplar leaves treated with SA andMeJA, salinity stress, low temperature, mechanical wounding, and infection with *M. brunnea* f.sp. *multigermtubi*, respectively. Approximately 1 μg of total RNA per sample was incubated with oligo (dT) beads to capture the polyadenylated RNA fraction. First-strand cDNA was synthesized using random hexamer-primer and reverse transcriptase (Invitrogen). The second-strand cDNA was synthesized using RNase H (Invitrogen) and DNA polymerase I (New England BioLabs). The cDNA libraries were then prepared according to protocols of Illumina. To summarize, one individual single-end cDNA library was constructed for each sample, and then sequenced on the Illumina GA platform for 35 cycles. The paired-end libraries were sequenced for 44–75bp.

The *P. trichocarpa* genome and annotated gene set were downloaded from the DOE Joint Genome Institute website (http://genome.jgi-psf.org/cgi-bin/), and 17 273 *P. trichocarpa* full-length cDNAs were collected from the reference genome sequence of *Populus* (version 2.0, Phytozome). The cDNAs were aligned to the *P. trichocarpa* genome and those with identities higher than 80% were retained for further analysis. After removing reads containing sequencing adapters and reads of low quality (containing Ns > 5), we aligned reads to the *P. trichocarpa* genome using SOAP (Li *et al.*, 2008), allowing for a 2bp mismatch between the tag and reference transcriptome. Reads that failed to be mapped were progressively trimmed off one base from the 39-end and mapped to the genome again until a match was found (*P*-value ≤ 0.05). For paired-end reads, the insert size was set between paired reads at 1bp to 10kb to allow reads spanning introns of different sizes. A similar strategy was used to align reads to the non-redundant gene set, but the insert length range was restricted to 1kb for paired-end read mapping.

#### Semi-quantitative RT-PCR analysis 

Total RNA from fresh tissues of poplar (*P. trichocarpa*) plants was extracted using RNA RNeasy PlantMiniKit (Qiagen, Germany) according to the manufacturer’s instructions with a modification as reported previously (Jia *et al.*, 2010). Samples from at least three plants were pooled for analysis. The total RNA before cDNA synthesis was treated with RNase-free DNase (TaKaRA, Dalian, China), according to the manufacturer’s instructions to avoid any genomic DNA contamination. First-strand cDNA was synthesized from 2 µg RNA with RT-AMV transcriptase (TaKaRa, Dalian, China) in a total volume of 20 μl by using oligo (dT)_18_ at 42°C for 30min. 18S rRNA was used as an internal control. The ampliﬁcation products of RT-PCR were resolved by 1% (w/v) agarose gel electrophoresis and visualized with ethidium bromide under UV light to test the expression levels of *PR* genes in transgenic plants.

#### Quantitative RT-PCR analysis 

Veriﬁcation of transcriptome data was conducted by checking the expression profiles of a number of the representative *PtrWRKY* genes in various tissues by quantitative RT-PCR (qRT-PCR) analysis. The gene-speciﬁc primers used for semi-qRT-PCR and qRT-PCR analysis are shown in Supplementary Table S3. RT-PCR analysis was based on at least two biological replicates of each sample and three technical replicates of each biological replicate.

### Cloning of *PtrWRKY89*

The full open-reading frame of *PtrWRKY89* was ampliﬁed with gene-speciﬁc primers (forward, 5′-AGTTCTTGACACCCACCACTC-3′; reverse, 5′- GGAAAATACAAAGAGGCTGC-3′; Joint Genome Institute, http://genome.jgi-psf.org/poplar/poplar.info.html) by RT-PCR with 2 μl cDNA from leaves. The PCR reaction was carried out with Pfu DNA polymerase (TaKaRa) in a total volume of 50 μl with an initial denaturing step at 94°C for 3min, 34 cycles of 94°C for 45 s, 54°C for 30 s, and 72°C for 90 s, and a ﬁnal extension step at 72°C for 10min. The ampliﬁcation products were cloned into the plant binary vector pCXSN, which is a zero-background TA cloning system that provides simple and high-efﬁciency direct cloning of PCR-ampliﬁed DNA fragments (Chen *et al.*, 2009*a*). The resulting vector p*35S:PtrWRKY89*, containing the *PtrWRKY89* open-reading frame under the control of the cauliﬂower mosaic virus (CaMV) 35S promoter and the hygromycin phosphortransferase gene (*Hpt*) as a plant-selectable marker conferring hygromycin resistance was transferred into *Agrobacterium tumefaciens* EHA105 by the freeze-thaw method.

### Transformation of *P. tomentosa* plants

*A. tumefaciens* strain EHA105 containing *35S:PtrWRKY89* was incubated in liquid yeast extract peptone medium supplemented with 100 mmol l^–1^ acetone-syringone at 18°C with constant shaking (200rpm) until the culture reached an optical density of 0.8 at OD_600_nm. The *A. tumefaciens* culture was then diluted with one volume of liquid woody plant medium (WPM) (Lloyd and McCown, 1980).

Poplar transformation methods were described previously by Jia *et al.* (2010). Leaves of *P. tomentosa* were excised from *in vitro* plantlets, cut into disks, and dipped in the diluted *Agrobacterium* culture for 8–10min. After excess liquid on the surfaces was absorbed by sterilized paper, the leaf disks were transferred to WPM medium [2.0mg l^–1^ zeatin, 1.0mg l^–1^ 1-naphthalene acetic acid (NAA)]. The infected disks were co-cultivated in the dark for 2 d and then transferred to callus-inducing medium containing 2.0mg l^–1^ zeatin, 1.0mg l^–1^ NAA, 400mg l^–1^ cefotaxime, 9mg l^–1^ hygromycin, and 0.8% (w/v) agar. After 2–3 weeks of culture in the dark, these leaf disks with induced calli were subcultured on screening medium [2.0mg l^–1^ zeatin, 0.1mg l^–1^ NAA, 400mg l^–1^ cefotaxime, 9mg l^–1^ hygromycin, and 0.8% (w/v) agar] to induce adventitious buds. Regenerated shoots were transferred to rooting medium containing 0.1mg l^–1^ NAA, 400mg l^–1^ cefotaxime, and 9mg l^–1^ hygromycin. Transgenic plants were selected with 9mg l^–1^ hygromycin. Rooted plantlets were acclimatized in pots placed inside a humid chamber (16h photoperiod, 25°C, 70% relative humidity) for 2 weeks and finally transferred to the greenhouse.

### Evaluation of transgenic plants for resistance against *D. gregaria*

To test the resistance of transgenic poplar against fungal infections, the *in vivo* test was performed with *D. gregaria* as described previously (Huang *et al.*, 2012). Adobe Photoshop was used to calculate lesion area. Each experiment was performed with at least three replicates, and contained wild-type controls. All data were analysed by *t*-test at *P* ≤ 0.05, using the Origin 6.1 software version v6.1052 (B232) (OriginLab Corp, Northhampton, MA, USA).

## Results

### Identification of poplar WRKY transcription factors and phylogenetic comparison of the WRKYs from different species

WRKY proteins comprise a large family of transcription factors and have been found in various plant species (Eulgem *et al.*, 2000; Xie *et al.*, 2005; Rensing *et al.*, 2008). In this study, a genome-wide analysis was carried out to identify *WRKY* genes in the *P. trichocarpa* genome using the publicly available genomic and putative full-length protein sequences, which were mainly downloaded from Phytozome v9.1 (http://www.phytozome.net/). Initially, we obtained 119 partial putative full-length protein sequences of *WRKY* genes in *P*. *trichocarpa* and their gene model IDs from the Plant Transcription Factor Database v2.0 (http://planttfdb.cbi.pku.edu.cn/). In an attempt to determine the reliability of these putative genes, the unique gene IDs for gene models were BLAST searched against Phytozome v6.0, resulting in a total of 105 members that were included in the WRKY superfamily identified above. Manual inspection of putative full-length protein sequences among these putative *WRKY* genes showed that three members contained only partial WDs whereas the other two members did not contain WDs or complete zinc finger motifs, and we removed both of them. The CDS sequences of 103 models were BLAST searched against Phytozome v9.1. Eventually we found a total of 100 members representing the unique poplar genes, and created consecutive nomenclature, designated as PtrWRKY1–PtrWRKY100 (Supplementary Table S1). These were used in the analysis.

**Fig. 1.**
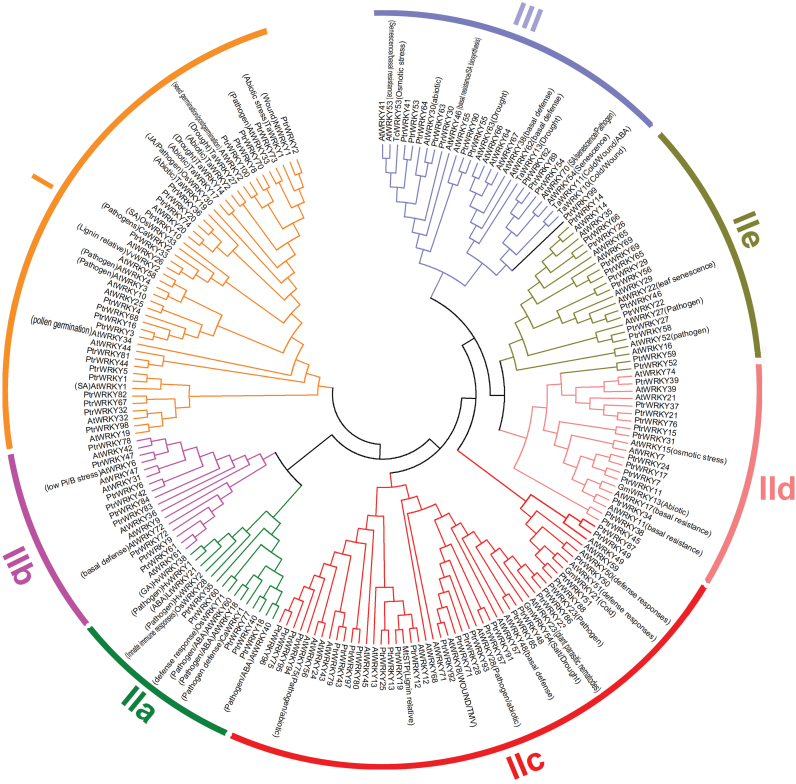
Phylogenetic tree of WRKY proteins from poplar, *Arabidopsis* and other species. The complete amino acid sequences of 100 poplar and 72 *Arabidopsis* WRKY proteins combined with 27 WRKY proteins from other plants were aligned by ClustalW, and the NJ tree was constructed using MEGA 4.1 with 1000 bootstrap replicates. Pathogen, pathogen response; Abiotic, abiotic stress response; SA, SA response; JA, JA response; ABA, ABA response; GA, GA response; TMV, tobacco mosaic virus response. This figure is available in colour at *JXB* online.

In general, transcription factor families contain a highly conserved domain or consensus motif involved in DNA binding, but the sequence similarity of other parts is relatively low in most genes (Wu *et al.*, 2005). A conserved DNA domain is considered as an evolutionary unit whose coding sequence can be duplicated and/or undergo recombination. The most prominent structural feature of the WRKY proteins is the WD of 60 amino acid residues (Eulgem *et al.*, 2000). All identified members of the *PtrWRKY* gene family contain either one or two WDs. In order to examine the phylogenetic relationships among *Populus* WRKY proteins, a multiple putative full-length protein sequence alignment of all putative 100 PtrWRKY proteins, 72 AtWRKYs from *Arabidopsis*, and 27 well-known WRKYs from other species was constructed using Clustal X (Thompson *et al.*, 1994, 1997) and an unrooted tree was built using MEGA 4.1 (Tamura *et al.*, 2007) by employing the NJ method. As shown in Fig. 1, this phylogram was classified into three groups (I, II, and III), based on the primary amino acid sequence. The WRKY members in group II can be further clustered into five subgroups (IIa–e) and a sole member, PtrWRKY99 (Fig. 1). Using the same method, the phylogenetic tree is also constructed based on WRKYdomainDs (Supplementary Figure S1). The members of group I contain two conserved WDs, an N-terminal WD (NT-WD) and a C-terminal WD (CT-WD), which are apparently clustered into two different clades (I-NT and I-CT), implying the retention of their divergence after duplication. The genes belonging to the NT-WDs and CT-WDs clades are assigned to IIa, IIb, and IIc, respectively, because they are obviously clustered into different clades. In addition, PtrWRKY99, which contains a C_2_H_2_ motif instead of C_2_HC, is clustered into subgroup III (Supplementary Figure S1).

### Variety of *cis*-elements in the promoter regions of *Populus WRKY* genes

**Fig. 2.**
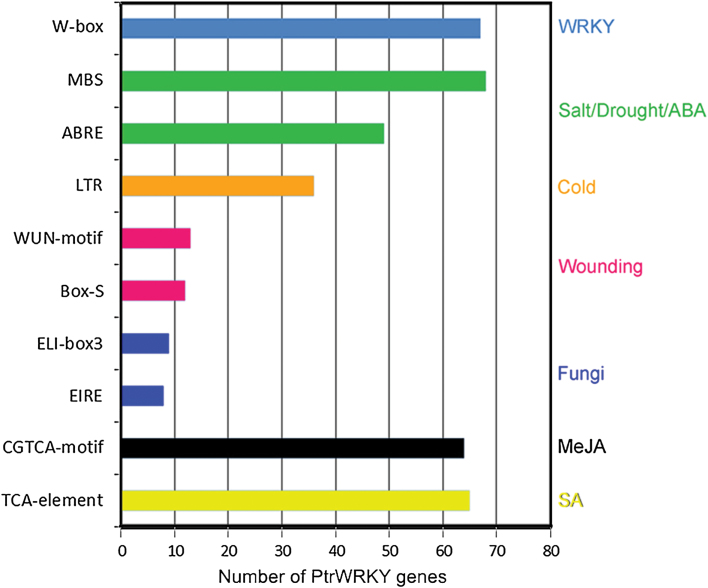
The number of WRKY genes containing various *cis*-acting elements. W-box, WRKY binding site; MBS, MYB binding site; ABRE, ABA-responsive element; LTR, *cis*-acting element involved in low-temperature responsiveness; WUN-motif: wound-responsive element; BOX-S, wounding responsive element; ELI-box3, elicitor-responsive element; EIRE, elicitor-responsive element; CGTCA-motif, *cis*-acting regulatory element involved in MeJA-responsiveness; TCA-element, *cis*-acting element involved in SA responsiveness. This figure is available in colour at *JXB* online.

Regulatory elements of the promoter sequences are essential to temporal, spatial, and cell type-specific control of gene expression (Lescot *et al.*, 2002). Here we searched 1500-bp upstream promoter regions of all putative *Populus WRKY* genes in the plant promoter database PlantCARE, and a number of *cis*-acting elements related to stresses as well as phytohormone responses were found. As shown in Fig. 2, SA-responsive elements (TCA-elements) and MeJA-responsive elements (CGTCA-motif) were found in the promoters of 65 and 64 *PtrWRKY* genes, respectively. Both of them existed in the promoter regions of 40 genes together. The conserved fungus-responsive elements, such as EIRE and ELI-box3, were present in the promoter regions of eight and nine *PtrWRKY*s, respectively. More than 12 promoters contained Box-S and WUN motifs involved in wounding stress. The low-temperature-responsive element (LTR) was found in 36 promoters of *PtrWRKY* genes. MYB-binding sites (MBSs) and ABA-responsive elements (ABREs) were abundant in *PtrWRKY* gene promoters and these elements were found in 68 and 49 promoters, respectively. In addition, WDs bound to W-boxes (TTGACC) with the highest afﬁnity; the invariant TGAC core of the W-box is essential for function and WRKY binding (Brand *et al.*, 2010; Brand *et al.*, 2013). There were several W-boxes in the promoters of 67 *PtrWRKY*s, indicating that these genes might be regulated by other WRKY proteins or themselves (Supplementary Table S4). These results indicated that the *Populus* WRKY transcription factors are involved in the transcriptional control of the defence and stress responses.

### Digital transcriptomics analysis of *Populus WRKY* genes

**Fig. 3.**
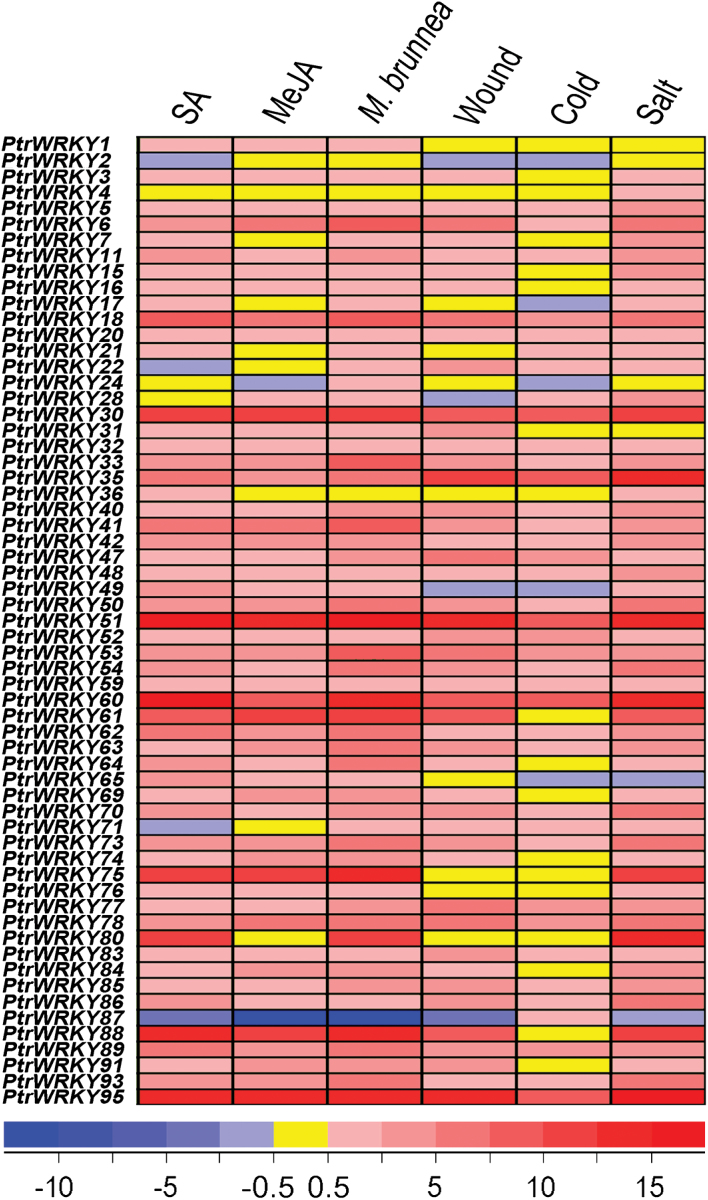
Digital transcriptomics analysis of *PtrWRKY* genes shows that 61 *PtrWRKY* genes are significantly sensitive to SA, MeJA, *M. brunnea* f.sp. *multigermtubi*, wounding, cold, and salinity stresses. Levels of transcript accumulation are shown. Each column represents a discreet biological sample. This figure is available in colour at *JXB* online.

To determine the potential roles of *PtrWRKY* genes in plant responses to various environmental stresses, induction experiments with several treatments were conducted with wild-type poplar plants. Total RNA was isolated from leaves of control, *M. brunnea*-infected, SA- and MeJA-treated, wound treated, low temperature-treated, and salinity treated plants, respectively. Global transcriptomics analysis revealed that transcript abundance of 61 *PtrWRKY* genes changed significantly after treatments compared to control plants (Fig. 3; more detailed information is listed in Supplementary Table S5). The majority of *PtrWRKY* genes were induced by both SA and MeJA, except for *PtrWRKY2*, *PtrWRKY22*, *PtrWRKY24, PtrWRKY71, PtrWRKY80* and *PtrWRKY87*. For example, *PtrWRKY51* and *PtrWRKY95*, belonging to group IIc, were activated by both SA and MeJA treatments, resulting in an obvious increase in mRNA level. Expression of *PtrWRKY80* was apparently increased in SA-treated plants, whereas no change in its expression was observed when treated with MeJA, indicating that *PtrWRKY80* could be only involved in the SA signalling pathway. In contrast, *PtrWRKY71* expression was downregulated significantly after SA treatment (Fig. 3). In addition, most of the *PtrWRKY* genes exhibited a gradual increase in expression levels in response to infection of the pathogen *M. brunnea* (Fig. 3 and Supplementary Table S5). Similar results were obtained in the genome-wide expression analysis of the *WRKY* gene superfamily in rice following SA- and MeJA-treatments, and pathogen infection (Ryu *et al.*, 2006). Meanwhile, 61 *PtrWRKY* genes were induced by wounding, low temperature, or salinity, except for *PtrWRKY1* (Supplementary Table S5). Transcript levels of *PtrWRKY75* and *PtrWRKY80* showed no change under mechanical wounding and low temperature treatments, but their mRNA levels were enhanced after salinity treatment. Expression of *PtrWRKY61* and *PtrWRKY88* increased after wounding and salinity treatments, but no change was detected under low temperature. In *Arabidopsis*, *AtWRKY18*, *AtWRKY40*, and *AtWRKY60* play important roles in plant responses to both abiotic and biotic stress (Chen *et al.*, 2010*a*; Wenke *et al.*, 2012), and their *Populus* orthologues, *PtrWRKY18, PtrWRKY35*, and *PtrWRKY60*, were transcriptionally upregulated by all treatments, indicating that these three *PtrWRKYs* are associated with responses towards biotic and abiotic stimuli. Based on the results presented here, we speculate that the functional divergence of PtrWRKY proteins plays a critical role in the responses of poplar plants to various stresses.

### Expression proﬁles of *Populus WRKY* genes in poplar

**Fig. 4.**
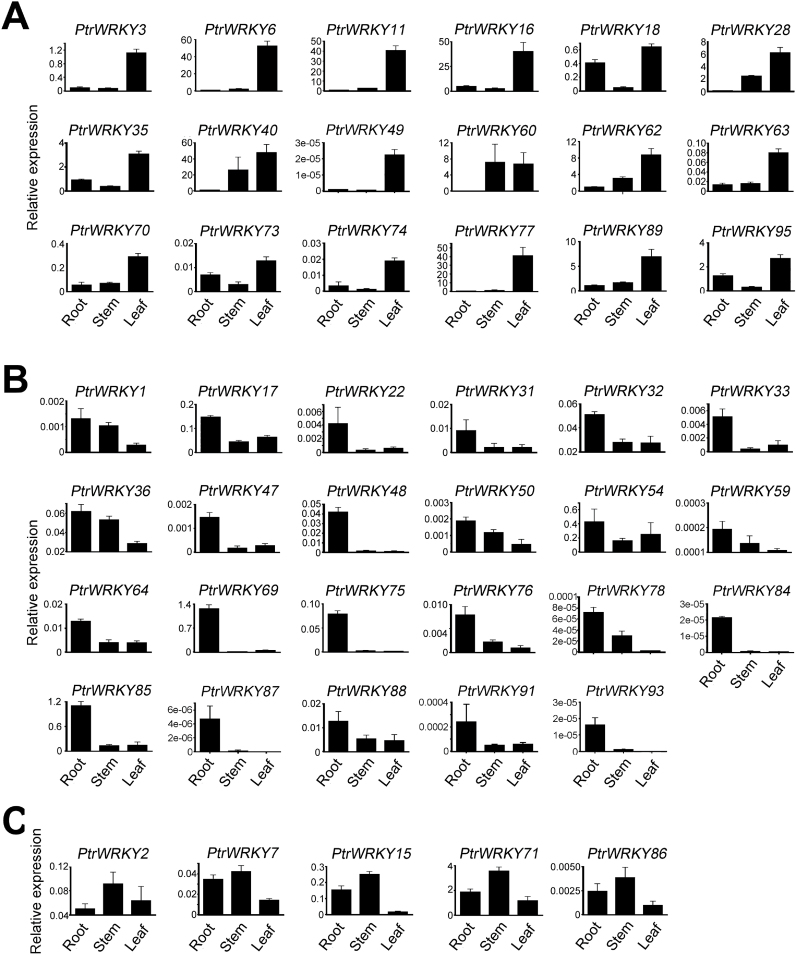
Expression analysis of 46 *PtrWRKY* genes using qRT-PCR. Relative quantities of *PtrWRKY* members in root, stem, and leaf are illustrated. (A–C) The members have highest expression levels in leaves, stems, and roots, respectively. Error bars result from three biological replicates. Poplar *18S* expression was used as a control and gene-speciﬁc primers were used for qRT-PCR analysis of *Populus WRKY* genes.

To investigate the tissue-speciﬁc expression of the *Populus WRKY* genes, qRT-PCR was used to determine the expression patterns of 61 *PtrWRKY* genes in various tissues including roots, stems, and leaves. Expression of only 46 *Populus WRKY* genes could be detected at the transcript level and these genes showed a diverse range of tissue-specific expression patterns in different tissues (Fig. 4). Most of *Populus WRKY* genes were more highly expressed in roots and leaves than in stems. Expressions of 14 *PtrWRKYs*, including *PtrWRKY3*, *6*, *11*, *16*, *28*, *35*, *49*, *62*, *63*, *70*, *74*, *77*, *89* and *95*, occur preferentially in leaves (Fig. 4A). Transcript levels of *PtrWRKY40* and *PtrWRKY60* were lower in roots but mRNA accumulation in stems and leaves was nearly similar. *PtrWRKY18* and *PtrWRKY73* showed higher expression levels in roots and leaves compare to stems. Both *PtrWRKY62* and *PtrWRKY89*, which are orthologous genes of *AtWRKY70* (Fig. 1), shared a similar expression patterns, indicating that they might play specific or redundant functional roles in poplar. For the other 23 *PtrWRKY* genes, high mRNA levels were observed in roots and relatively low but unambiguous expression was evident in stems and leaves (Fig. 4B). Additionally, five *PtrWRKY* genes, including *PtrWRKY2, PtrWRKY7, PtrWRKY15, PtrWRKY71* and *PtrWRKY86,* were mainly expressed in stems (Fig. 4C).

### Expression patterns of 18 *Populus WRKY* genes in response to different treatments

**Fig. 5.**
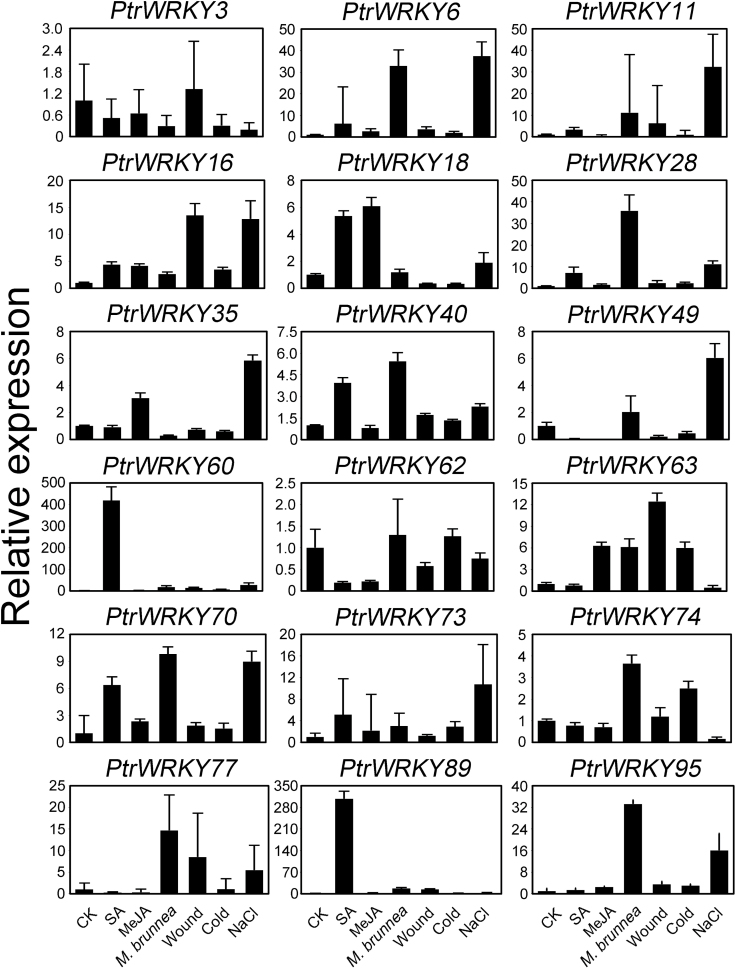
Expression profiles of 18 *Populus WRKY* genes under different treatments. Leaves excised from poplar were sprayed with SA and MeJA, inoculated with *M. brunnea* f.sp. *multigermtubi*, and treated with wounding, cold, and salinity stresses. The untreated leaves were regarded as the control (CK). Leaves were collected for transcript profile analysis by qRT-PCR. Error bars result from three biological replicates. Poplar *18S* expression was used as a control and gene-speciﬁc primers were used for qRT-PCR analysis of *Populus WRKY* genes.

To further confirm if the expression of *Populus WRKY* genes was induced by different biotic and abiotic stresses, 18 *PtrWRKY* members, whose mRNA levels were relatively high in leaves, were selected and qRT-PCRs were performed to analyse their expression patterns in response to these treatments. Overall, transcript levels of all *PtrWRKY* genes tested were detected to respond to at least one treatment (Fig. 5). Among them, three *PtrWRKY* genes were significantly induced by only one treatment. For instance, *PtrWRKY28* responded to *M. brunnea*, whereas SA induced a remarkable increase in mRNA levels of *PtrWRKY60* and *PtrWRKY89*. Several *PtrWRKY* genes, such as *PtrWRKY6*, *PtrWRKY16*, *PtrWRKY18*, *PtrWRKY35*, *PtrWRKY40*, *PtrWRKY62* and *PtrWRKY74*, were able to respond to two treatments. For example, *PtrWRKY18* was induced by both SA and MeJA, indicating that it might be a node of convergence for JA-mediated and SA-mediated signal transduction pathways. The expression of *PtrWRKY40* was enhanced significantly after treatments with SA and *M. brunnea* (Fig. 5), implying that its role might be involved in pathogen resistance mediated by an SA signal.

### Identification and sequence analysis of the *PtrWRKY89* gene

**Fig. 6.**
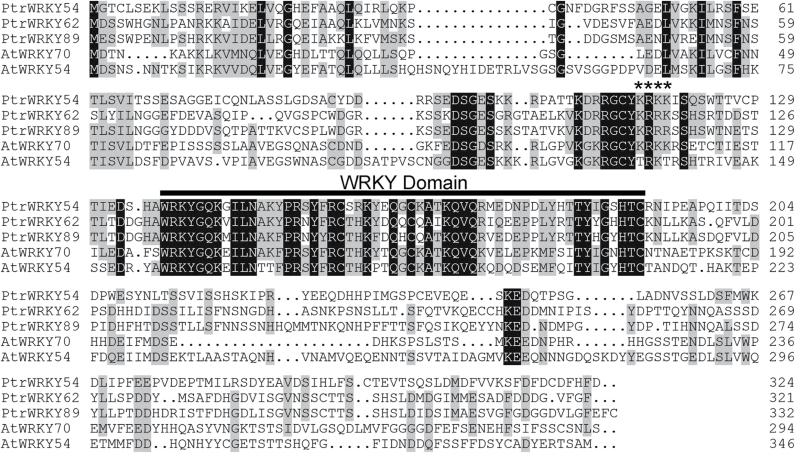
Comparison of PtrWRKY89-deduced amino acid sequence with PtrWRKY54, PtrWRKY62, AtWRKY54, and AtWRKY70 proteins. Identical amino acids are indicated by white letters on a black background, and conserved amino acids by black on a grey background. Asterisks indicate the nuclear localization signals. The WD is underlined. Putative full-length protein sequences were aligned with the DNAMAN program.

As shown in Fig. 1, PtrWRKY89 has been classiﬁed into group III of the WRKY family and formed a subgroup with PtrWRKY54, PtrWRKY62, AtWRKY54 and AtWRKY70, whose domains are distinct from those of monocot species (He *et al.*, 2012). To elucidate the role of *PtrWRKY89* in SA signalling, we identified the *PtrWRKY89* cDNA encoding a putative WRKY protein by RT-PCR with gene-specific primers based on the sequences deposited in Phytozome version 9.1. *PtrWRKY89* appeared to be a full-lengh cDNA of 1002bp encoding a protein of 333 amino acids residues. PtrWRKY89 contains a typical WD with a C_2_HC-type zinc finger and putative nuclear localization signals. Interestingly, the putative full-length protein sequence of PtrWRKY89 exhibits low similarity to AtWRKY70 (48.38%) (Fig. 6), but their WDs were conserved (81.09%) (Fig. 6). Previous studies demonstrated that almost all WRKY III proteins were responsive to SA treatment (Kalde *et al.*, 2003). Among them, AtWRKY38, AtWRKY46, AtWRKY53, AtWRKY54, AtWRKY62 and AtWRKY70 were early SA-induced, with highest expression levels 2h after SA treatment (Besseau *et al.*, 2012). As shown in Fig, 1, PtrWRKY89 belongs to the same cluster as AtWRKY54 and AtWRKY70 characterized in *Arabidopsis* (Knoth *et al.*, 2007). These results indicate that PtrWRKY89 is a potential transcriptional activator in the SA signalling pathway and is induced by SA at an early stage.

### *PtrWRKY89* is induced at an early stage by SA treatment

**Fig. 7.**
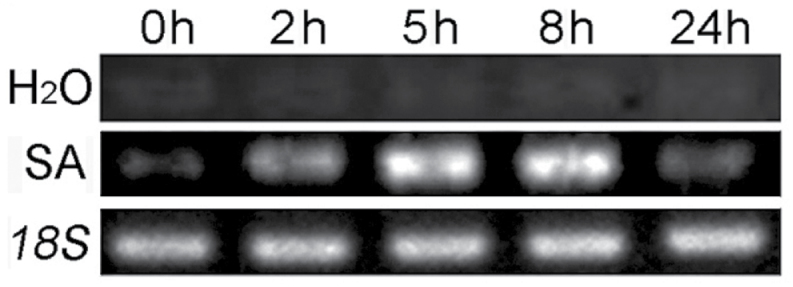
Induction profiles of *PtrWRKY89*. Leaves from poplar were sprayed with SA. Control plants were sprayed with H_2_O. Leaves were collected after 0, 2, 5, 8, and 24h for transcript profile analysis by semi-qRT-PCR. Poplar *18S* expression was used as a control and gene-speciﬁc primers were used for semi-qRT-PCR analysis of *PtrWRKY89.*

The expression pattern of *PtrWRKY89* was analysed by semi-qRT-PCR at different time points when treated with SA. As shown in Fig. 7, expression of *PtrWRKY89* was not induced by mock treatment. While the mRNA level of *PtrWRKY89* increased 4-fold up to 2h after exposure to SA, the highest transcript level was detected from 5–8h. However, transcript accumulation decreased significantly after 24h of SA treatment. These data indicate that *PtrWRKY89* is an early gene activated by the SA signal.

### Overexpression of *PtrWRKY89* confers increased resistance to *D. gregaria* in transgenic poplar

To further investigate the roles of *PtrWRKY89* in plant biotic stress responses, a plant binary construct containing a full-length *PtrWRKY89* cDNA driven by the CaMV 35S promoter was generated and transformed into *P. tomentosa* by *A. tumefaciens*-mediated transformation. A total of 17 putative transformants with hygromycin resistance were obtained and grown in the greenhouse. No obvious phenotypic change was observed in transgenic plants when compared to wild-type plants (Supplementary Figure S3). PCR analysis using gene-speciﬁc primers was employed to confirm the presence of the transgenes in transformed plants. An expected ampliﬁcation product of a 1202-bp PtrWRKY89 fragment combining a T-NOS terminal region was obtained from all transgenic lines tested, whereas no signal was detected from untransformed plants (Supplementary Figure S4), indicating the successful integration of the transgene into the poplar genome. From all independent transgenic lines containing the *35S*:*PtrWRKY89* construct, two lines (LC and LI) with high transcript levels of *PtrWRKY89* were selected for further analysis.

**Fig. 8.**
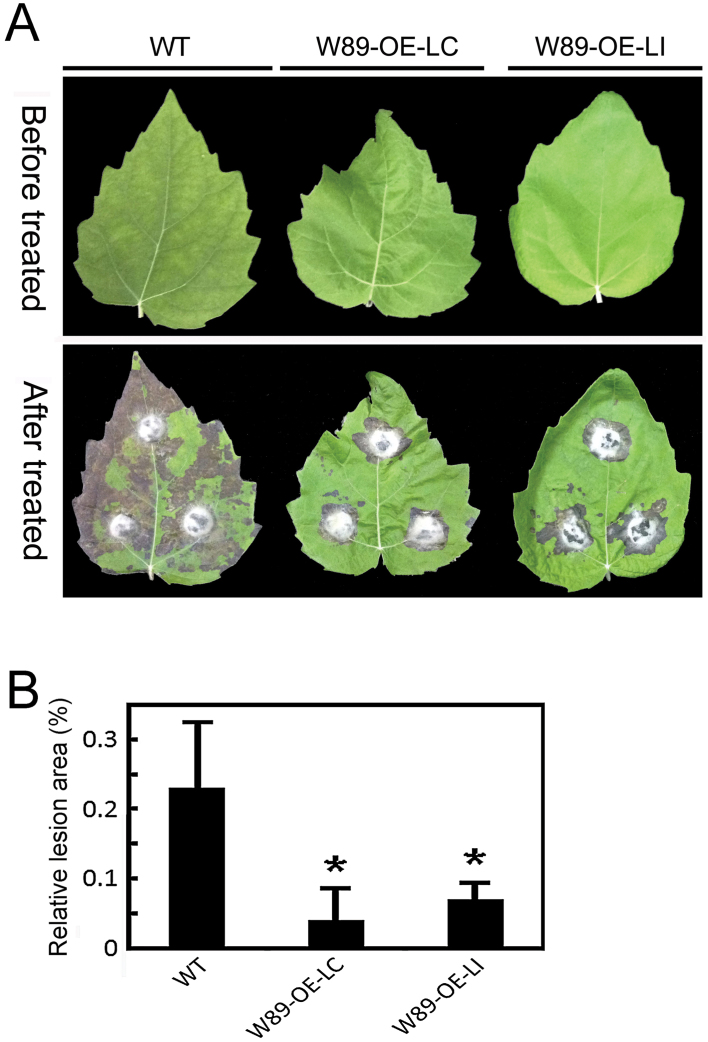
Resistance of transgenic poplar plants inoculated with *D. gregaria*. (A) The leaves from wild-type and transgenic plants before treatment and after infection with *D. gregaria* 3 d after inoculation were photographed. (B) Mean infected area of transgenic lines to the fungal pathogen; *PtrWRKY89* confers resistance to *D. gregaria* in transgenic poplar plants. Values are means of three replications. Error bars indicate standard deviations. Asterisks indicate a statistically signiﬁcant difference between wild-type and transgenic plants (*P* < 0.05 by student’s *t*-test). This figure is available in colour at *JXB* online.

To determine the effect of *PtrWRKY89* overexpression on disease resistance in poplar, leaves excised from transgenic and control lines were inoculated with agar plugs containing hyphae of *D. gregaria*, a hemibiotrophic fungus. Compared with the severe disease symptoms appeared on the control leaves at 3 d post inoculation (dpi), only slight necrotic lesions appeared on the leaves of the transgenic *35S*:*PtrWRKY89* lines tested (Fig. 8A). Quantiﬁcation assays showed that the lesions were signiﬁcantly (*P* < 0.05) smaller in *35S:PtrWRKY89* lines than in the control plants (Fig. 8B), indicating that *PtrWRKY89* might act as a positive regulator of basal resistance to infection of hemibiotrophic fungal pathogens.

### Constitutive expression of the *PtrWRKY89* gene in poplar resulted in the upregulation of several *PR* genes

**Fig. 9.**
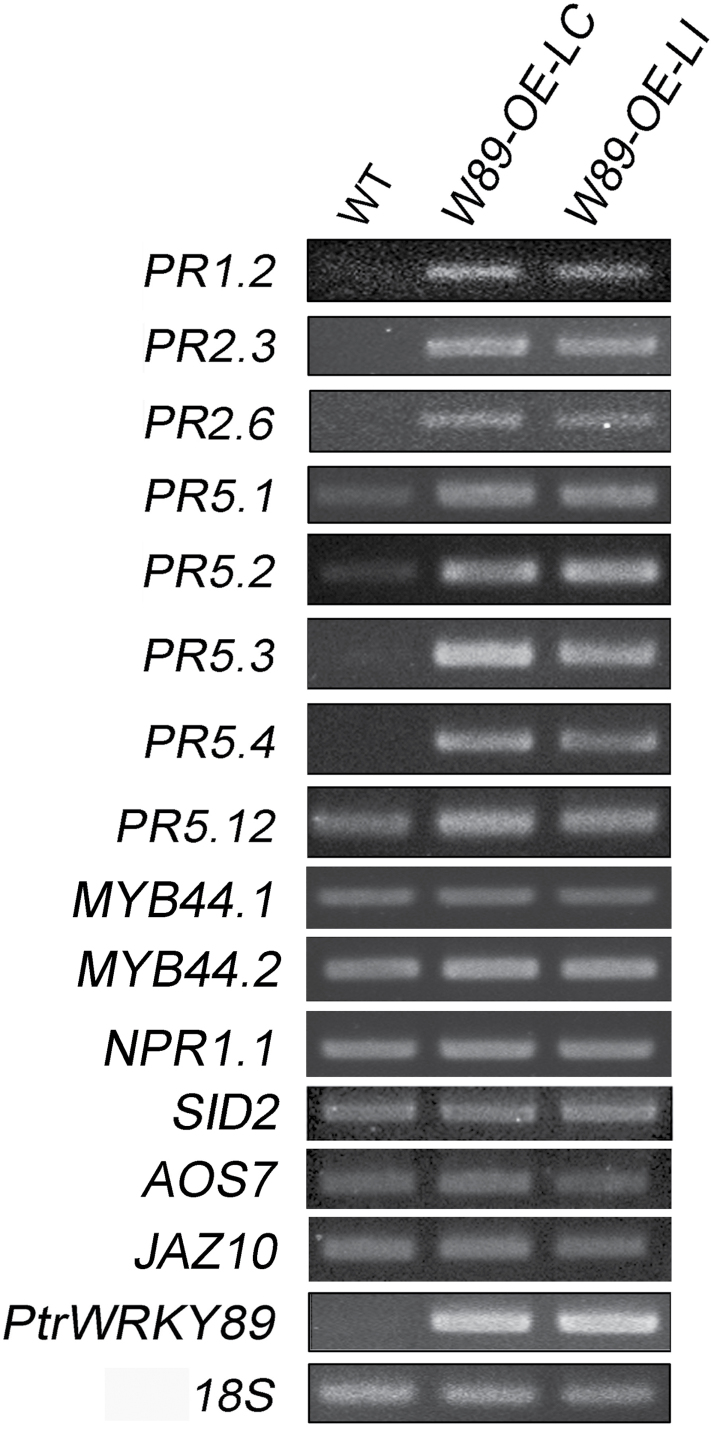
Gene expression analyses of defence-related genes in plants overexpressing *PtrWRKY89*. Transcript levels of PR genes, *MYB44.1, MYB44.2, NPR1.1, SID2, AOS7,* and *JAZ10* in wild-type and transgenic plants were elevated by semi-qRT-PCR. Poplar *18S* expression was used as a control.

Increased disease resistance in plants is often accompanied by the accumulation of elevated transcript levels of *PR* genes associated with the SA-mediated defence pathway (Uknes *et al.*, 1992; Dong, 2004; Loake and Grant, 2007). Because exogenous SA-triggered expression of *PtrWRKY89* and overexpression of *PtrWRKY89* enhanced resistance to pathogens, we further determined whether *PtrWRKY89* is directly involved in controlling expression of *Populus PR* genes. A genome-wide candidate gene screen found 15 putative *Populus PR* genes, homologues to *PR1, PR2*, and *PR5* from *Arabidopsis*. Semi-qRT-PCR analysis showed that eight out of 15 *PR* genes, including *PR1.2*, *PR2.3*, *PR2.6*, and *PR5*s, were activated in *PtrWRKY89*-overexpressing lines (Fig. 9). Interestingly, the expression of other *Populus PR* genes was not upregulated in *PtrWRKY89*-overexpressing plants. In addition, the mRNA levels of *NPR1.1*, *MYB44.1*, *MYB44.2*, and *SID2* showed no remarkable differences between wild-type and transgenic plants (Fig. 9). Similarly, *AOS7* and *JAZ10*, involved in the JA-signalling pathway, showed no change (Fig. 9). Taken together, these results indicate that *PtrWRKY89* might be one of the coactivators of SA-mediated defence marker genes in poplar.

## Discussion

### Characterization of the *Populus WRKY* gene family

In a previous study, Zhu *et al.* (2007) proposed the existence of 104 WRKY family genes in the *P. trichocarpa* genome using the conserved WDs as the deﬁning feature. He *et al.* (2012) further presented detailed information regarding the specifics of the individual *Populus WRKY* genes. However, in our study, only 100 members of the WRKY transcription factor superfamily were found in the *P. trichocarpa* genome. A new member, POPTR_0017s14630.1, was identified (Supplementary Table S1) and two members (POPTR_0003s20860.1 and POPTR_0810s00200.1) described by He *et al.* (2012) were removed in our database because they could not be found in the upgraded database; we also identified three members (POPTR_0014s08600.2, POPTR_0015s11130.2, and POPTR_0001s47670.2) that should be considered as alternative transcripts of POPTR_0014s08600.1, POPTR_0015s11130.1, and POPTR_0001s47670.1, respectively.

### Classification and phylogenetic analysis of *WRKY* genes in *Populus*, *Arabidopsis* and other species

Phylogenetic comparison of the WRKY proteins has been conducted extensively in *Arabidopsis*, rice, canola, and poplar, and the evolutionary relationships of this gene family within and among the different species has been intensively studied (Eulgem *et al.*, 2000; Xie *et al.*, 2005; Yang *et al.*, 2009*b*; He *et al.*, 2012). In *Arabidopsis* and rice, the WRKY members were classified into three large groups (I, II, and III) based on the number of the WD and the features of their zinc-finger-like motifs (Wu *et al.*, 2005). However, group I contained two subgroups, Ia and Ib: members of subgroup Ia harboured two WDs; subgroup Ib contained these proteins with a single WD. Additionally, on the basis of the phylogenetic results, group II was divided into four subgroups (IIa–d) and group III divided into subgroups IIIa and IIIb (Wu *et al.*, 2005). In *Populus*, three major groups were categorized as described by Wu *et al.* (2005). Several subgroups, such as Ia, Ib, and IIa–e, were clearly formed on the basis of the phylogenetic analysis (He *et al.*, 2012). An alternative method also divided *Arabidopsis* WRKY members into three big groups (I, II, and III) (Eulgem *et al.*, 2000). In detail, the members with two WDs with C_2_H_2_ zinc fingers belonged to group I, the members of group II encoded proteins containing a single WD and C_2_H_2_-type zinc finger, split up into five distinct subgroups (IIa–e) and the single WDs of the proteins including C_2_HC zinc fingers belonged to group III. However, further studies indicated that the spacing of the zinc fingers was responsible for the diversity of WRKYs (Zhang and Wang., 2005; Brand *et al.*, 2013). For example, subgroup IIc contained C-X_4_-C-X_22–23_-HXH. Subgroup IIa, IIb, IId, and IIe shared C-X_5_-C-X_23_-HXH and group III contained the C-X_4–7_-C-X_23-(24–30)_-HXC pattern (Brand *et al.*, 2013). According to the results reported by Eulgem *et al.* (2000) and Brand *et al.* (2013), we obtained an overall phylogenetic tree of the WRKY proteins from *Arabidopsis*, *Populus* and other species. Among the 100 putative PtrWRKY proteins, 22 members with two WDs and C_2_H_2_ zinc fingers belonged to group I. Most (68) *PtrWRKY* genes, which encoded proteins containing a single WD and C_2_H_2_-type zinc finger, belong to group II and there was also a sole member (PtrWRKY99) (Fig. 1 and Supplementary Figure S1). Generally, the WDs of group I and II members have the same type of zinc finger motif (C_2_H_2_), whose pattern of potential zinc ligands (C_2_H_2_) is unique among all zinc-like-motifs described (Eulgem *et al.*, 2000). However, these WRKY members containing a C_2_HC motif, instead of a C_2_H_2_ pattern, were classified into group III (Eulgem *et al.*, 2000). In *Populus*, 10 WRKY proteins with C_2_HC zinc fingers together with one WD belong to group III (Fig. 1). Interestingly, phylogenetic analyses revealed that PtrWRKY99 was clustered into subgroup III. However, this member has a typical C_2_H_2_-type zinc finger of group II (Eulgem *et al.*, 2000). Based on the report by Brand *et al.* (2013), PtrWRKY99, which contained a C-X_4_-C-X_21_-HXH motif, should be assigned to group II but as a sole member (Fig. 1; Supplementary Figures S1 and S2). In addition, based on the conserved WDs, PtrWRKY49 and PtrWRKY87 with high similarity were separated from subgroup IId, IIe, and group III (Supplementary Figure S1). This finding was inconsistent with the results shown in Fig. 1, in which these two members and AtWRKY49 were clustered with subgroup IIc. In an attempt to further confirm the classification of PtrWRKY49 and PtrWRKY87, sequence alignment of their WDs was conducted. The result showed that WDs of these two members contained a C-X_4_-C-X_23_-HXH zinc finger (Supplementary Figure S2), indicating that PtrWRKY49 and PtrWRKY87 were typical members of subgroup IIc.

A phylogenetic tree combining transcription factor families from different species will not only help our understanding of the phylogenetic relationships among the members, but also allow speculation on the putative functions of the proteins based on the functional clades identiﬁed (Zhang *et al.*, 2012). For instance, TaMYB32 was selected out from 60 isolated wheat MYB genes and demonstrated to enhance the tolerance to salt stress in transgenic *Arabidopsis* (Zhang *et al.*, 2012). Because the functions of several WRKY proteins have been well characterized experimentally, phylogenetic analysis allowed the identification of several functional clades. However, it was obvious that some members had various or overlapping functions (Fig. 1). For example, AtWRKY18, AtWRKY40, and AtWRKY60 were involved in pathogen and ABA responses simultaneously (Chen *et al.*, 2010*a*), while HvWRKY38, belonging to group IIa, responded to gibberellin (GA) (Zou *et al.*, 2008). In the clade of group III, AtWRKY53 was related to senescence and basal resistance (Miao and Zentgraf, 2007); however, TcWRKY53 was associated with the osmotic stress response (Niu *et al.*, 2012). Additionally, PtrWRKY54, PtrWRKY62, and PtrWRKY89 could be assigned to a clade with AtWRKY54 and AtWRKY70 (Fig. 1), which seemed to have similar roles. However, *PtrWRKY62* and *PtrWRKY89* had different expression patterns (Fig. 5). These results imply that it is arbitrary to divide clades of the WRKY family according to their motif sequence similarity without further experiment evidence.

### *Populus* WRKY proteins respond to biotic and abiotic stresses

Plants have had to adopt different strategies to respond to various biotic and abiotic stresses for survival in adverse environmental conditions (Ahuja *et al.*, 2010). Increasing research has indicated that WRKY proteins play vital roles in the regulation of gene expression to deal with environmental change (Ülker and Somssich, 2004; Eulgem and Somssich, 2007; Rushton *et al.*, 2010). In plants, response to stress needs several signalling molecules including SA, JA, abscisic acid (ABA) and ethylene (ET). The expression levels of *WRKY* genes changed rapidly after hormone treatments (Chen and Chen, 2002; Li *et al.*, 2004; Lai *et al.*, 2008; Yang *et al.*, 2009*a*; Ramiro *et al.*, 2010) In *Populus*, CGTCA-motifs and TCA elements randomly distributed in the promoter regions of *PtrWRKY* genes (Fig. 2 and Supplementary Table 4), implying that most *PtrWRKY* genes were involved in SA and JA responses. *PtrWRKY60* and *PtrWRKY89* were significantly induced by SA (Fig. 5), indicating that they functioned as key factors in regulating specific signalling pathways. *WRKY* genes were also directly induced by pathogens in plants, such as *AtWRKY18* (Chen and Chen, 2002), *CaWRKY2* (Oh *et al.*, 2006), *AtWRKY70* (Knoth *et al.*, 2007), *PtWRKY23* (Levée *et al.*, 2009), *AtWRKY3*, and *AtWRKY4* (Lai *et al.*, 2008). Many *PtrWRKY* genes contained EIRE and ELI-box3 elements in their promoters and 59 members were induced after inoculation of *M. brunnea* (Fig. 3). Additionally, WRKY transcription factors were associated with responses to abiotic stresses. For instance, *TaWRKY2* and *TaWRKY19* regulate abiotic stress tolerance in transgenic *Arabidopsis* plants (Niu *et al.*, 2012). Expression of *GsWRKY20* in *Arabidopsis* enhances drought tolerance and regulates ABA signalling (Luo *et al.*, 2013). Overexpression of *WRKY25* or *WRKY33* was sufﬁcient to increase *Arabidopsis* NaCl tolerance and increasing sensitivity to ABA (Jiang and Deyholos, 2009). *GmWRKY13*, *GmWRKY21*, and *GmWRKY54* confer differential tolerance to abiotic stresses in transgenic *Arabidopsis* plants (Zhou *et al.*, 2008). Wounding-induced *AtWRKY8* functions antagonistically with its interacting partner VQ9 to modulate salinity stress tolerance (Chen *et al.*, 2010*b*; Hu *et al.*, 2013). In *Populus*, 10 *WRKY* genes, belonging to group III, were induced by varieties of stresses, such as cold, salinity, and drought, but no further analysis was performed (He *et al.*, 2012). In this work, among the 100 *PtrWRKY* genes, 60 members responded differentially to at least one treatment including wounding, cold, and salt, except for *PtrWRKY1* (Fig. 3), and at least eight *PtrWRKY* genes were significantly induced by salinity stress (Fig. 5). Overall, the results reveal that most *PtrWRKY* genes are involved in responses to multiple biotic and abiotic stresses, consistent with previous studies (Eulgem and Somssich, 2007).

### Different expression trajectories between *PtrWRKY89* and its paralogue

In *planta*, some genes and their paralogues had constantly redundant functions: AtWRKY18, AtWRKY40, and AtWRKY60 (Xu *et al.*, 2006); AtWRKY11 and AtWRKY17 (Journot-Catalino *et al.*, 2006); AtWRKY54 and AtWRKY70 (Besseau *et al.*, 2012; Li *et al.*, 2013); and AtWRKY3 and AtWRKY4 (Lai *et al.*, 2008). However, expression patterns of these paralogous genes might not always be identical. In *Arabidopsis*, *AtWRKY53* was more sensitive to SA treatment than its paralogue *AtWRKY41* (Besseau *et al.*, 2012). The synergistic effect of losing *AtWRKY11* and *AtWRKY17* function in untreated plants implied that they acted in a partially redundant manner as repressors of *AtWRKY70*, but not *AtWRKY54* (Journot-Catalino *et al.*, 2006). In addition, *AtWRKY4* could be induced by *B. cinerea* but the expression level of *AtWRKY3* was not affected (Lai *et al.*, 2008). In *Populus*, we also noticed that *PtrWRKY62* and *PtrWRKY89* had different expression trajectories (Fig. 5). Like AtWRKY54 and AtWRKY70, PtrWRKY62 and PtrWRKY89 are paralogues but still showed sequence divergence outside the WD. Therefore, we speculate that these two members had redundant roles, but diverse functions surely exist in *Populus*.

### Constitutive expression of *PtrWRKY*89 enhanced resistance to fungal pathogens

In plants, plenty of WRKY proteins played important roles in defence responses by mediating SA, JA, ethylene and ABA signalling. Ectopic expression of some *WRKY* genes changed the transduction of hormone signalling and then altered tolerance of transgenic plants to biotic stresses. For example, transgenic poplars overexpressing and underexpressing *PtWRKY23* were both more susceptible to *Melampsora* infection than wild-type plants (Levée *et al.*, 2009). In *Arabidopsis*, single WRKY mutants of *AtWRKY18*, *AtWRKY40*, and *AtWRKY60* exhibited no or small alterations in response to the pathogens. However, *wrky18/wrky40* and *wrky18/wrky60* double mutants and the *wrky18/wrky40/wrky60* triple mutant were substantially more resistant to *P. syringae* but more susceptible to *B. cinerea* than untransformed plants (Xu *et al.*, 2006). Overexpression of *AtWRKY70* did not affect endogenous levels of free SA in *Arabidopsis* (Li *et al.*, 2004). However, *AtWRKY70* was downstream of *NPR1* and *MYB44* in an SA-dependent signal pathway and acted as an activator of SA-induced genes such as *PR1*, *PR2*, and *PR5*. Meanwhile it was also a repressor of JA-responsive genes. Hence overexpression of *AtWRKY70* could enhance resistance to hemibiotrophic pathogens and make plants more susceptible to necrotrophic fungal pathogens (Li *et al.*, 2004; Shim *et al.*, 2013). In our study, *PtrWRKY89* were significantly induced by SA, indicating its potential role in SA-mediated signalling pathways (Fig. 5). Overexpression of *PtrWRKY89* did not enhance the level of *SID2* mRNA involved in SA synthesis (Fig. 9), indicating the stability of free SA in transgenic poplars compared with the wild type. Additionally, *PtrWRKY89* was constitutively expressed in transgenic poplar plants, resulting in an increased resistance to hemibiotrophic fungal pathogen *D. gregaria* (Fig. 8). Moreover, expression of *PR* genes, as a molecular marker of the SA signalling pathway, was activated in *PtrWRKY89* overexpressors (Fig. 9). These results indicate that PtrWRKY89 enhanced resistance to *D. gregaria* via the activation of *PR* genes but not improvement of SA levels. Our findings are partially consistent with a previous report in *Arabidopsis* WRKY70 (Li *et al.*, 2004). In addition, we also noticed that overexpression of *PtrWRKY89* did not change the levels of *AOS7* and *JAZ10*, which were involved in the JA signalling pathway. However, it is still unknown what the precise function of PtrWRKY89 is in the JA pathway in *Populus.* Overall, our work provided useful information for improving the resistance and stress tolerance of woody plants and suggested that PtrWRKY89 plays a regulatory role in he SA signalling pathway to increase poplar defence.

## Supplementary material

Supplementary data can be found at *JXB* online.

Supplementary Table S1. Common names and gene IDs of putative PtrWRKYs in different versions of Phytozome.

Supplementary Table S2. Common names of WRKYs from different species and their accession numbers in GenBank.

Supplementary Table S3. Primers for qRT-PCR and semi-qRT-PCR in this study.

Supplementary Table S4. Various *cis*-acting elements responsive to stresses in WRKY promoters.

Supplementary Table S5. Results of DGE analysis.

Supplementary Figure S1. Phylogenetic analyses of *Populus* WDs.

Supplementary Figure S2. Multiple sequence alignment of WDs using DNAMAN.

Supplementary Figure S3. Transgenic *P. tomentotosa* plants overexpressing *PtrWRKY89*.

Supplementary Figure S4. PCR analysis of transgenic poplar plants.

## Funding

This work was supported by the National Natural Science Foundation of China (31370672, 31171620), the National Key Project for Research on Transgenic Plants (2011ZX08010-003), 100 Talents Programme of The Chinese Academy of Sciences, the Natural Science Foundation Project of CQ CSTC (CSTC2013JJB8007), the programme for New Century Excellent Talents in University (NCET-11–0700) and the Research Fund for the Doctoral Programme of Higher Education (20110182110004).

## Supplementary Material

Supplementary Data
