# Supplementary material for: Genome-wide identification and characterization of the Populus WRKY transcription factor family and analysis of their expression in response to biotic and abiotic stresses
Source: J Exp Bot. 2014 Sep 23;65(22):6629–44. doi: 10.1093/jxb/eru381 (PMC4246191; doi:10.1093/jxb/eru381)
Supplement: Supplementary Data [file supp_65_22_6629__index.html]

Genome-wide identification and characterization of the Populus WRKY transcription factor family and analysis of their expression in response to biotic and abiotic stresses — Genome-wide identification and characterization of the Populus WRKY transcription factor family and analysis of their expression in response to biotic and abiotic stresses — Supplementary Data 

# Genome-wide identification and characterization of the *Populus* WRKY transcription factor family and analysis of their expression in response to biotic and abiotic stresses

## Supplementary Data

Data files

**Files in this Data Supplement:**

- Supplementary Data - Supplementary Data
