# Supplementary material for: Genome-wide identification and characterization of the Populus WRKY transcription factor family and analysis of their expression in response to biotic and abiotic stresses
Source: J Exp Bot. 2014 Sep 23;65(22):6629–44. doi: 10.1093/jxb/eru381 (PMC4246191; doi:10.1093/jxb/eru381)
Supplement: Supplementary Data [file supp_eru381_jexbot130336_file001.pdf]

Genome-wide identification and characterization of the *Populus* WRKY transcription factor family and analysis of their expression in response to biotic and abiotic stresses

Authors:

Yuanzhong Jiang[1], Yanjiao Duan[1], Jia Yin[3], Shenglong Ye[1], Faqi Zhang[3], Wanxiang Lu[2], Di Fan[1], Keming Luo*[1,3]

[1-]Key Laboratory of Eco-environments of Three Gorges Reservoir Region, Ministry of Education, Chongqing Key Laboratory of Transgenic Plant and Safety Control, Institute of Resources Botany, School of Life Sciences, Southwest University, Chongqing 400715, China

[2-]College of Horticulture and Landscape Architecture, Southwest University, Chongqing, 400716, China

[3-]Key Laboratory of Adaptation and Evolution of Plateau Biota, Northwest Institute of Plateau Biology, Chinese Academy of Sciences, 810008 Xining, China

*Corresponding author (kemingl@swu.edu.cn).

Address: No. 1, Tiansheng Road, Beibei, Chongqing 400715, China.

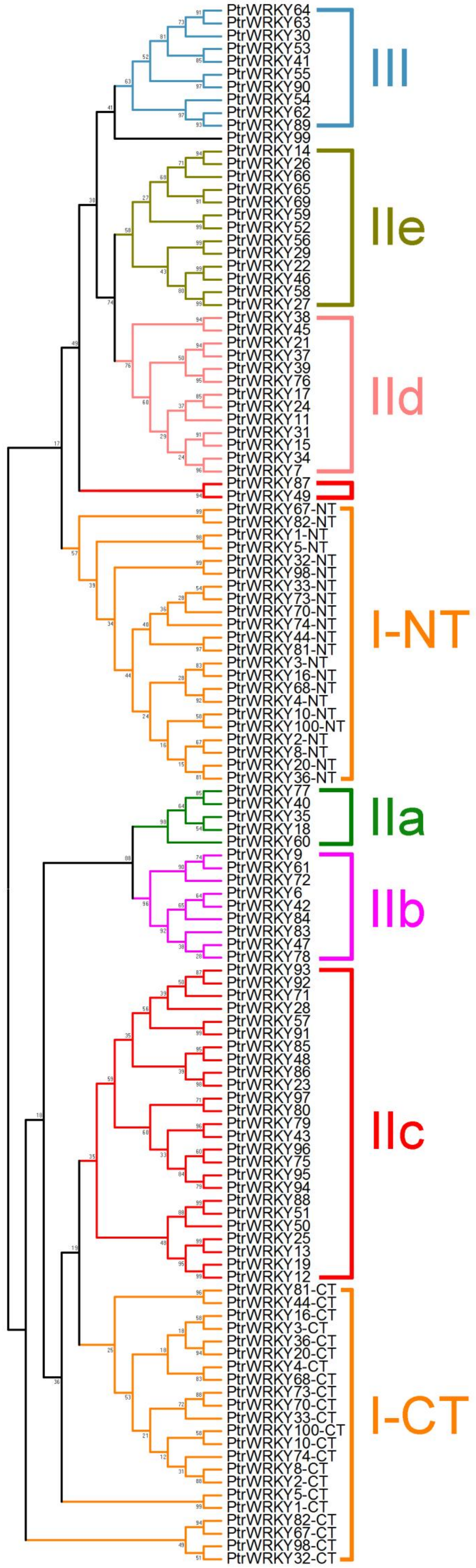

Supplementary Fig. S1. Phylogenetic analyses of *Populus* WRKY domains. The majority rule consensus phylogenetic unrooted tree was reconstructed based on the sequences 122 *Populus* WRKY domains using the MEGA 4.0 program by the Neighbor-Joining (NJ) method with 1,000 bootstrap replicates. Group I-NT and -CT domains (orange); group IIa domains (green); group IIb domains (purple), group IIc domains (red); group IId domains (pink); group IIe domains (khaki); sole domain (black); group III domains (mauve).

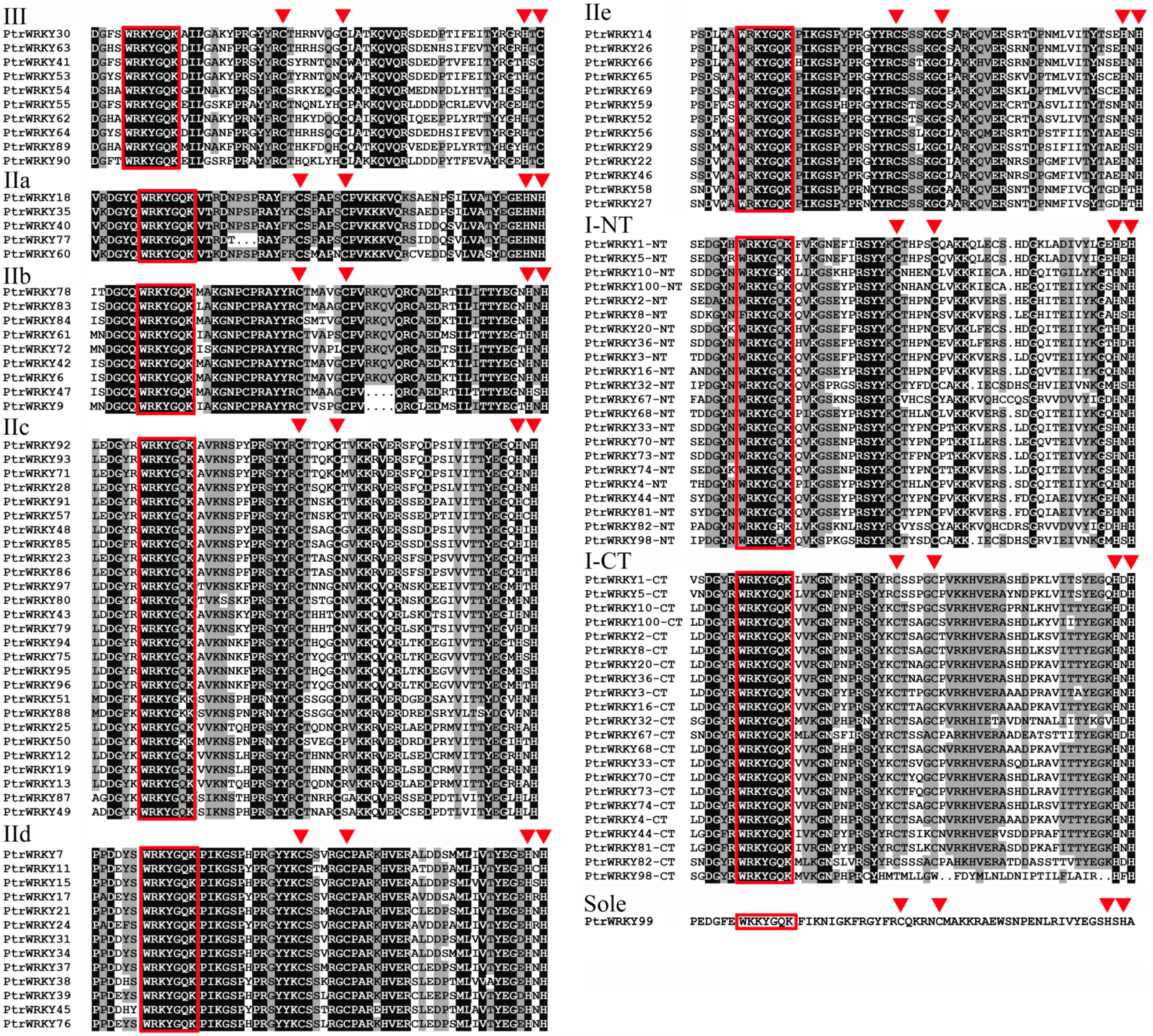

Supplementary Fig. S2. Multiple sequence alignment of WRKY domains using DNAMAN. One hundred PtrWRKY proteins were divided into three groups based on the features of their WRKY domains. Groups II was further divided into subgroups (IIa, IIb, IIc, IId, IIe). In addition, PtrWRKY99 was regarded as a sole. Red boxes housed the WRKYGQK motif and the zinc-finger motif sequences were highlighted by red triangles.

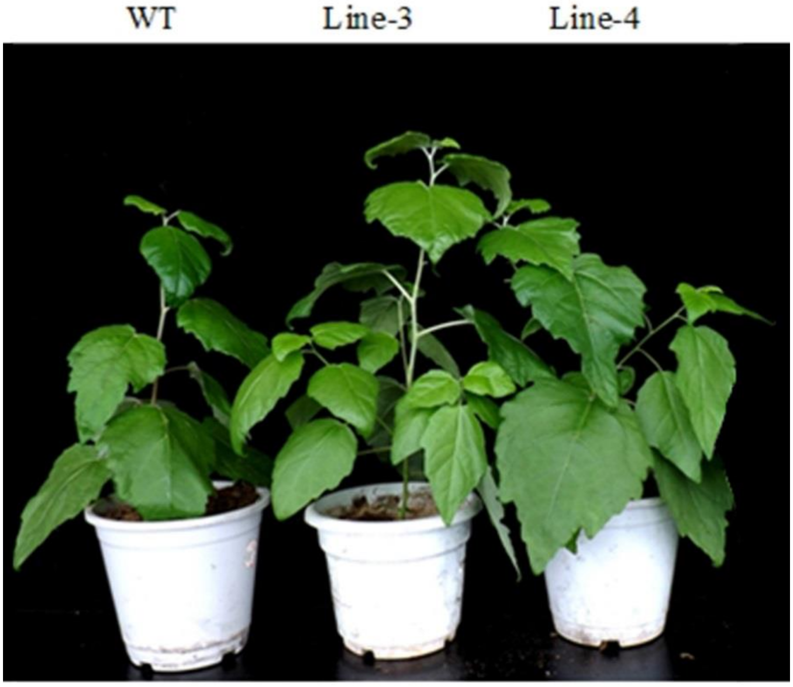

Supplementary Fig. S3. Transgenic *Populus tomentosa* Carr. plants overexpressing *PtrWRKY89*. No phenotypic or growth differences were observed between *PtrWRKY89* overexpressors and control plants under the same growth conditions.

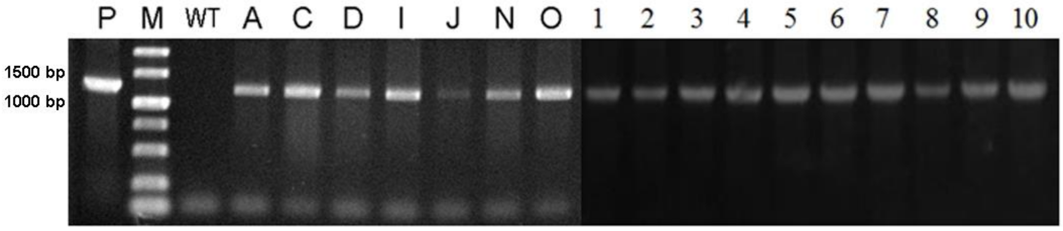

Supplementary Fig. S4. PCR analysis of transgenic poplar plants. Genomic DNAs were isolated from hygromycin-resistant plants transformed with the 35S:*PtrWRKY89* vector. PCR amplification used primers specific for the production of a 1202-bp PtrWRKY89 fragment combining T-NOS terminal region. M, D2000 DNA Ladder; WT, non-transgenic plants; P, corresponding plasmid DNA (positive control); Numbers on the left indicate DNA marker sizes in base pairs.

Supplemental Table S1. WRKY gene family in *Populus*						
common name	sequence ID VISION 3.0	common name (He et al., 2012)	sequence ID (VISION 2.0)	accession number	AtWRKY homologue	length(aa)
PtrWRKY1	Potri.014G164300	PtWRKY2	POPTR_0014s16220.1	ERP53352.1	1	485
PtrWRKY2	Potri.011G169300	PtWRKY37	POPTR_0011s17230.1	XP_002330532	2	725
PtrWRKY3	Potri.008G091900	PtWRKY69	POPTR_0008s09140.1	XP_002312267	3	492
PtrWRKY4	Potri.017G088300	PtWRKY87	POPTR_0017s12430.1	EEF04212.1	58	453
PtrWRKY5	Potri.002G221600	PtWRKY54	POPTR_0002s22180.1	XP_002302839	1	316
PtrWRKY6	Potri.004G007500	PtWRKY28	POPTR_0004s00890.1	XP_002331543	42	578
PtrWRKY7	Potri.002G123300	PtWRKY14	POPTR_0002s12480.1	XP_002301166	15	358
PtrWRKY8	Potri.001G472800	PtWRKY101	POPTR_0001s47670.1	XP_002300596	2	731
PtrWRKY9	Potri.001G208600	PtWRKY42	POPTR_0001s21580.1	XP_002298189	9	519
PtrWRKY10	Potri.019G053900	PtWRKY92	POPTR_0019s08110.1	ERP49067.1	25	501
PtrWRKY11	Potri.018G008500	PtWRKY24	POPTR_0018s03450.1	XP_002324382	11/17	338
PtrWRKY12	Potri.002G138900	PtWRKY48	POPTR_0002s13980.1	EEE80510.2	12	203
PtrWRKY13	Potri.005G086400	PtWRKY10	POPTR_0005s08860.1	ABK93467.1	13	164
PtrWRKY14	Potri.001G460600	PtWRKY45	POPTR_0001s46490.1	XP_002299016	35	423
PtrWRKY15	Potri.005G141400	PtWRKY20	POPTR_0005s18490.1	ERP61327.1	7	331
PtrWRKY16	Potri.010G163000	PtWRKY72	POPTR_0010s17040.1	XP_002315024	3/4	499
PtrWRKY17	Potri.006G072400	PtWRKY7	POPTR_0006s07170.1	XP_002308998	11/17	301
PtrWRKY18	Potri.006G263600	PtWRKY9	POPTR_0006s27950.1	XP_002308704	18/60	320
PtrWRKY19	Potri.014G050000	PtWRKY95	POPTR_0014s04890.1	XP_002327027	12	175
PtrWRKY20	Potri.011G087900	PtWRKY73	POPTR_0011s09030.1	EEE97371.2	20	395
PtrWRKY21	Potri.005G219500	PtWRKY63	POPTR_0005s24100.1	XP_002306823	21	347
PtrWRKY22	Potri.002G164400	PtWRKY49	POPTR_0002s16590.1	ERP64413.1	22	409
PtrWRKY23	Potri.014G118200	PtWRKY33	POPTR_0014s11350.1	XP_002320967	23	290
PtrWRKY24	Potri.018G139300	PtWRKY91	POPTR_0018s13600.1	XP_002325248	11/17	300
PtrWRKY25	Potri.007G078200	PtWRKY68	POPTR_0007s06930.1	XP_002310044	13	233
PtrWRKY26	Potri.011G157100	PtWRKY74	POPTR_0011s16050.1	XP_002317080	14	450
PtrWRKY27	Potri.017G149000	PtWRKY96	POPTR_0017s00960.1	XP_002327503	27	412
PtrWRKY28	Potri.002G059100	PtWRKY47	POPTR_0002s06000.1	XP_002302140	N/A	325
PtrWRKY29	Potri.001G099000	PtWRKY41	POPTR_0001s09900.1	ERP65337.1	29	325
PtrWRKY30	Potri.012G031700	PtWRKY27	POPTR_0012s01380.1	XP_002318373	30	371
PtrWRKY31	Potri.007G047400	PtWRKY26	POPTR_0007s10670.1	XP_002310122	7	335
PtrWRKY32	Potri.018G107000	PtWRKY30	POPTR_0018s11630.1	EEF03700.1	32	427
PtrWRKY33	Potri.016G128300	PtWRKY17	POPTR_0016s13600.1	XP_002323637	33	579
PtrWRKY34	Potri.014G024200	PtWRKY94	POPTR_0014s02420.1	ERP52684.1	15	388
PtrWRKY35	Potri.018G019800	PtWRKY89	POPTR_0018s02470.1	XP_002324327	18/60	320
PtrWRKY36	Potri.001G361600	PtWRKY44	POPTR_0001s37260.1	XP_002298853	20	557
PtrWRKY37	Potri.002G043500	PtWRKY46	POPTR_0002s04440.1	XP_002302070	21	351
PtrWRKY38	Potri.003G111900	PtWRKY55	POPTR_0003s11120.1	XP_002304424	N/A	310
PtrWRKY39	Potri.005G055300	PtWRKY98	POPTR_0005s05700.1	XP_002328486	39/74	353
PtrWRKY40	Potri.001G044500	PtWRKY88	POPTR_0001s03620.1	XP_002332076	40	318
PtrWRKY41	Potri.001G092900	PtWRKY12	POPTR_0001s10490.1	XP_002297983	41/53	338
PtrWRKY42	Potri.014G111900	PtWRKY79	POPTR_0014s10750.1	XP_002320254	42	502
PtrWRKY43	Potri.014G090700	PtWRKY19	POPTR_0014s08640.1	EEE98475.2	43	117

PtrWRKY44	Potri.006G133200	PtWRKY93	POPTR_0006s13550.1	XP_002326326	26	475
PtrWRKY45	Potri.001G121300	PtWRKY22	POPTR_0001s00350.1	EEE82428.2	N/A	314
PtrWRKY46	Potri.014G090300	PtWRKY78	POPTR_0014s08600.1	ERP53148.1	22	349
PtrWRKY47	Potri.002G228400	PtWRKY53	POPTR_0002s21330.1	XP_002302873	6/31	613
PtrWRKY48	Potri.008G103300	PtWRKY70	POPTR_0008s10280.1	EEE89685.2	48	368
PtrWRKY49	Potri.006G087000	PtWRKY32	POPTR_0006s08730.1	EEE92589.2	49	304
PtrWRKY50	Potri.006G224100	PtWRKY67	POPTR_0006s24050.1	XP_002308538	50	165
PtrWRKY51	Potri.005G085200	PtWRKY61	POPTR_0005s08720.1	XP_002307134	51	204
PtrWRKY52	Potri.014G119800	PtWRKY80	POPTR_0014s11490.1	XP_002320972		262
PtrWRKY53	Potri.003G138600	PtWRKY21	POPTR_0003s13840.1	XP_002304549	41/53	342
PtrWRKY54	Potri.013G090300	PtWRKY77	POPTR_0013s09470.1	XP_002319879	54/70	324
PtrWRKY55	Potri.019G059300	PtWRKY36	POPTR_0019s08730.1	EEE99872.2	55	351
PtrWRKY56	Potri.003G132700	PtWRKY56	POPTR_0003s13250.1	XP_002303610	29	330
PtrWRKY57	Potri.010G160100	PtWRKY71	POPTR_0010s16760.1	EEF02268.2	57	293
PtrWRKY58	Potri.004G072000	PtWRKY59	POPTR_0004s07050.1	EEE85782.2	27	418
PtrWRKY59	Potri.002G195300	PtWRKY3	POPTR_0002s19630.1	EEE80787.2	N/A	245
PtrWRKY60	Potri.018G019700	PtWRKY38	POPTR_0018s02480.1	XP_002324328		271
PtrWRKY61	Potri.015G064100	PtWRKY99	POPTR_0015s07530.1	XP_002330089	9	565
PtrWRKY62	Potri.016G137900	PtWRKY85	POPTR_0016s14490.1	XP_002323675	54/70	321
PtrWRKY63	Potri.002G168700	PtWRKY51	POPTR_0002s17010.1	XP_002302620	30	363
PtrWRKY64	Potri.014G096200	PtWRKY6	POPTR_0014s09190.1	XP_002320852	30	365
PtrWRKY65	Potri.011G070100	PtWRKY8	POPTR_0011s06990.1	XP_002317397	69	268
PtrWRKY66	Potri.017G104800	N/A	POPTR_0017s14630.1	KJ569571	35	237
PtrWRKY67	Potri.006G264000	PtWRKY15	POPTR_0006s27990.1	ERP59906.1	32	267
PtrWRKY68	Potri.004G120800	PtWRKY60	POPTR_0004s12000.1	XP_002306024	58	482
PtrWRKY69	Potri.004G060900	PtWRKY58	POPTR_0004s05920.1	XP_002305730	69	262
PtrWRKY70	Potri.019G123500	PtWRKY4	POPTR_0019s14460.1	XP_002326093	33	599
PtrWRKY71	Potri.005G203200	PtWRKY62	POPTR_0005s22480.1	XP_002306743	71	322
PtrWRKY72	Potri.017G079500	PtWRKY86	POPTR_0017s11570.1	XP_002323839	72	538
PtrWRKY73	Potri.013G153400	PtWRKY1	POPTR_0013s14960.1	XP_002319959	33	591
PtrWRKY74	Potri.006G105300	PtWRKY64	POPTR_0006s10600.1	ERP59048.1	26	561
PtrWRKY75	Potri.T043800	PtWRKY97	POPTR_0017s09750.1	ERP51013.1	75	186
PtrWRKY76	Potri.013G042600	PtWRKY25	POPTR_0013s03990.1	EEE95005.2	39/74	354
PtrWRKY77	Potri.003G182200	PtWRKY5	POPTR_0003s18060.1	EEE78831.2	40	316
PtrWRKY78	Potri.011G007800	PtWRKY100	POPTR_0021s00280.1	XP_002317127	42	590
PtrWRKY79	Potri.002G164900	PtWRKY50	POPTR_0002s16640.1	EEE80650.2	43	125
PtrWRKY80	Potri.015G099200	PtWRKY82	POPTR_0015s11130.1	XP_002322274	56/24	178
PtrWRKY81	Potri.016G083600	PtWRKY84	POPTR_0016s08440.1	ERP51650.1	26	382
PtrWRKY82	Potri.018G019000	PtWRKY90	POPTR_0018s02550.1	EEF02898.1	32	271
PtrWRKY83	Potri.014G155100	PtWRKY81	POPTR_0014s15320.1	XP_002321134	6/31	624
PtrWRKY84	Potri.002G186600	PtWRKY52	POPTR_0002s18770.1	XP_002302808	N/A	506
PtrWRKY85	Potri.010G147700	PtWRKY31	POPTR_0010s15750.1	EEF01133.2	48	374
PtrWRKY86	Potri.002G193000	PtWRKY13	POPTR_0002s19390.1	EEE80797.2	23	318
PtrWRKY87	Potri.016G099900	PtWRKY34	POPTR_0016s10610.1	XP_002323509	49	309
PtrWRKY88	Potri.007G079800	PtWRKY16	POPTR_0007s06720.1	XP_002310610	51	190
PtrWRKY89	Potri.006G109100	PtWRKY65	POPTR_0006s10950.1	XP_002309186	54/70	333

PtrWRKY90	Potri.013G090400	PtWRKY76	POPTR_0013s09460.1	EEE95268.2	55	349
PtrWRKY91	Potri.008G094000	PtWRKY11	POPTR_0008s09360.1	EEE88696.2	57	293
PtrWRKY92	Potri.011G079300	PtWRKY103	POPTR_0011s02790.1	ERP55043.1	71	331
PtrWRKY93	Potri.001G352400	PtWRKY29	POPTR_0001s34520.1	ERP66462.1	71	312
PtrWRKY94	Potri.001G058800	PtWRKY40	POPTR_0001s13600.1	XP_002299321	75	104
PtrWRKY95	Potri.003G169100	PtWRKY18	POPTR_0003s16750.1	EEE79720.2	75	157
PtrWRKY96	Potri.001G328000	PtWRKY43	POPTR_0001s33560.1	EEE84925.2	75	186
PtrWRKY97	Potri.012G101000	PtWRKY75	POPTR_0012s10290.1	XP_002318747	56/24	186
PtrWRKY98	Potri.006G184800	PtWRKY66	POPTR_0006s19850.1	ERP59684.1	32	345
PtrWRKY99	Potri.001G002400	PtWRKY39	POPTR_0001s03970.1	ERP65099.1	N/A	135
PtrWRKY100	Potri.013G086000	PtWRKY35	POPTR_0013s08380.1	XP_002328969	25	536
		PtWRKY23	POPTR_0014s08600.2	XP_002320158	22	322
		PtWRKY57	POPTR_0003s20860.1	XP_002303982	N/A	116
		PtWRKY83	POPTR_0015s11130.2	N/A	56/24	125
		PtWRKY102	POPTR_0001s47670.2	N/A	2	724
		PtWRKY104	POPTR_0810s00200.1	N/A	71	319

Supplementary Table S1. The common names and the gene IDs of putative PtrWRKYs in different visions of Phytozome. The nomenclature by He et al (2009) was also listed. Additionally, The putative orthologs in Arabidopsis of their respective WRKY domain sequences, the accession numbers in GenBank and the length of protein were shown (He et al., 2009). N/A presented not available

Supplemental Table S2. Accession Numbers of WRKYs from Different Species	
name	accession number
CaWRKY2	DQ402421
OsWRKY71	AB190817
TcWRKY53	ABN79278
TaWRKY10	EU665439
TaWRKY11	EU665440
TaWRKY13	EU665426
GmWRKY13	NP_001237376
GmWRKY54	NP_001237438
TaWRKY19	EU665430
TaWRKY17	EU665429
TaWRKY2	EU665425
TaWRKY14	EU665427
TaWRKY27	EU665431
GmWRKY21	NP_001237327
OsWRKY33	AAF23898
TaWRKY1	EU665424
PtWRKY23	EF051079
CaWRKY1	AAX20040
HvWRKY1	AAS48544
HvWRKY2	CAH68818
NtWRKY1	AB022693
HvWRKY38	AY541586
LtWRKY21	AY792618
OsWRKY28	DAA05093
OsWRKY30	HM153428
VvWRKY2	AY596466
MtSTP	HM622066

Supplementary Table S2. Common names of WRKYs from different species and their accession numbers in GenBank.

Supplemental Table S3. Primers for quantitative real-time RT-PCR (qRT-PCR) and semi-qRT-PCR		
For Gene	F	R
PtrWRKY1	5’-GGTGTTGAAGAAAGTCATG-3’	5’-GAGTAGAGCTGTCATTACA-3’
PtrWRKY2	5’-AATCCATCTTCCTCGGTA-3’	5’-CAACTTTCCTTCTCTTGG-3’
PtrWRKY3	5’-TGAGGGCTTTGGAATGAT-3’	5’-AGGTCAAAAACTGTGTCG-3’
PtrWRKY4	5’-AAGAGAAGGCAATGCAGG-3’	5’-AGCAGCAGGGACATCATG-3’
PtrWRKY5	5’-GACCTGTCCACTGTTGAA-3’	5’-AGTAGCTCCTGGGATTTG-3’
PtrWRKY6	5’-ATTGCCTCAGGTTTTTGGTC-3	5’-ATTTGGGGTTGAGCTTGGTG-3’
PtrWRKY7	5’-CTCTTCTTCTGGTCGCTG-3’	5’-ACATCCTCTCACGCTGCT-3’
PtrWRKY11	5'-AGGGTTCCAGCAATAAGTTC-3’	5'-TCATCTGTTGCTCTCTCCAC-3'
PtrWRKY15	5’-TTCCAGCAATCAGCTTGA-3’	5’-GTAGTAACCTCTTGGATG-3’
PtrWRKY16	5'-TCTCCTCAGGGCTTTGGAA-3'	5'-AAGGTCGAAGACATTGCCG-3'
PtrWRKY17	5’-GGAAGCTGTCATTGCTCC-3’	5’-CCTCTTGGGTATGGTGAG-3’
PtrWRKY18	5’-TTATGAAGGAGAGCACAACC-3’	5’-TTCTGATGGATGATGGACTG-3’
PtrWRKY20	5’-GATCATCCTAAACCTCAACC-3’	5’-CATCCAACCTCCTGCTTC-3’
PtrWRKY21	5’-GTCCTTGAGTATTGATGG-3’	5’-ACCCTATGTTTCCTCTTC-3’
PtrWRKY22	5’-TCTCACACACCTCGATCC-3’	5’-CTCTGCTGTGTACGTCAC-3’
PtrWRKY24	5’-AAAGAAGGAAATCAAGGGTG-3’	5’-TAGGGGAGCATGTGAGTG-3’
PtrWRKY28	5’-TCGACATCAATAGGGCAGAG-3’	5’-CCATAATCAGGAGAATAGAGC-3’
PtrWRKY30	5’-AAGAAAGAGCATGCCGAGA-3’	GAGAGGAAGCAAATTAGAGG
PtrWRKY31	5’-CCTCCTTTATCTACATCTTC-3’	5’-CATCTTTCTGCTTTTCTTGG-3’
PtrWRKY32	5’-GAAGGGAAACTTAGAATGC-3’	5’-ATAGTTCCTGGGATGAGG-3’
PtrWRKY33	5’-TCTAATGGTCTTGCATCTCC-3’	5’-GGATTGAAAGAAGGATGATG-3’
PtrWRKY35	5’-TTATGAAGGAGAGCACAACC-3’	5’-CAAATCAAGGGTCACAGTAG-3’
PtrWRKY36	5’-CTTCATCTCTGCCTAACC-3’	5’-AGCATCCAACCTCCTTCT-3’
PtrWRKY40	5’-GTCAAGAAGAAGGTTCAAAGG-3’	5’- GTGAGGTCGAGAGTAATATTG-3’
PtrWRKY41	5’-CAAAGAAGAGAAAGACCAC-3’	5’-TTCCATGAGCACAAGAGT-3’
PtrWRKY42	5’-GCATAAACACCGGATTG-3’	5’-TCCCCTTTCTGCTCTATC-3’
PtrWRKY47	5’-GCTGACAAGAAACATGATGT-3’	5’-CAGAACTGCTAGCTCACTCT-3’
PtrWRKY48	5’-AGATCAAGAGGAAGAGCAG-3’	5’-GTGCAACGATAATAACTCC-3’
PtrWRKY49	5’-AACAAGCCCAAGAGTACTCG-3’	5’-GAACGGTAAGATGAAGATGAGG-3’
PtrWRKY50	5’-AAGTGGTGGAGAGGGTGAAG-3’	5’-TAGTAATTCCTCGGGTTTGG-3’
PtrWRKY51	5’-GCATGCAAAATGGAGTGAAG-3’	5’-TAGTTATCACATAAGCCGAG-3’
PtrWRKY52	5’-CAAATCAGAAGAGAACAATG-3’	5’-AAGAAGGGACAACAGGAATC-3’
PtrWRKY53	5’-CAAAGAAGAGAAAGACCAC-3’	5’-GTGATCTCAAATATGGTGG-3’
PtrWRKY54	5’-AGTTACTTTAGGTGTTCTCG-3’	5’-AGAACTGATCACACTACTCG-3’
PtrWRKY59	5’-AAGAAGGAAGGTGGTTGAG-3’	5’-GGATGGTTGTGATTTGAGG-3’
PtrWRKY60	5’-GTCAAGAAGAAGGTCCAAAG-3’	5’-TGATGAGGTATGTGGAGAAC-3’
PtrWRKY61	5’-GGAAACAGGTACAAAGATG-3’	5’-GAAGATGACAGTGAAGAG-3’
PtrWRKY62	5’-TCGAACAAGCCAAGCAACTC-3’	5’-CATGATACCATCCATGTCCAG-3’
PtrWRKY63	5’-TAGTCCTTGGAGTGAAGTC-3’	5’-GCATCTGTAGTATCCTCTTG-3’
PtrWRKY64	5’-ACAGTAGTCCTAGGAGTG-3’	5’- GCATCTGTAGTATCCTCTTG-3’
PtrWRKY65	5’-CGAAGTAAGAAGGCCATGC-3’	5’- TTGTGGGATCTAGAAGGAG-3’
PtrWRKY69	5’-AGATGGCTTCCACTTCCTC-3’	5’- ATAGTATCCT CTGGGGTAAG-3’
PtrWRKY70	5’-AATCCAAGGAGCTACTAC-3’	5’-GTTACCATTGTTGTTGTGG-3’
PtrWRKY71	5’-AGAAGGCAGTGAAGAATAGC-3’	5’-CCAGAAGGGAAGGTGATAGC-3’

PtrWRKY73	5'-ATGGCTGCTTCTTCAGGGAGC-3'	5'-ACTACTCTTCCAGTTCAAACCC-3'
PtrWRKY74	5’-AAGATGGCTGGGACAGAACG-3’	5’-GTAGCTCCTT GGATTTGG-3’
PtrWRKY75	5’-ATTGGGTCTCCAACAGAG-3’	5’-ATCGGTAATAGCTCCTCGG-3’
PtrWRKY76	5’-GTCATCCACAAGATCATTC-3’	5’-CTCAGTTTCCTCCTCTTTG-3’
PtrWRKY77	5’-ATGTCAGGACTGAAGCTG-3’	5’-CGATACTCCTTTGAACCT-3’
PtrWRKY78	5’-GGAAGCTCCCATGATTACTG-3’	5’-CATTGCAGCTGGAGGTAGAG-3’
PtrWRKY80	5’-CTAGTAGAATCTGTAACG-3’	5’- GTAGTAACTTCTTGGAAAC-3’
PtrWRKY83	5’-TTGCTGCTGATCCTAACTTC-3’	5’-CATTGCTATTGCTGTTGCTG-3’
PtrWRKY84	5’-TGCTCCATGACTGTAGGATG-3’	5’-TAAGAGCATTGTTGCTGCAG-3’
PtrWRKY85	5’-CTTCTATCTCTTCCTCTTCG-3’	5’-GTACTTCCTCCACCTAAACC-3’
PtrWRKY86	5’-CAGAGGTGTTGAATCAGC-3’	5’- GCAACGATAGTAACTCCTAGG-3’
PtrWRKY87	5’-CGCCTTCCAAGAAACCTAAG-3’	5’-GGAGGAAGATGGTGAAGTAGG-3
PtrWRKY88	5’-GCATGCAAAATGGAGTGAAG-3’	5’-TGGGCTGTTCTTGACTGAC-3’
PtrWRKY89	5’-TCCAACGATCCACAATAACC-3’	5’-TAAAACATCACCGCCGTCTC-3’
PtrWRKY91	5’-TGCTAGTCACATGACTCCTCC-3’	5’-AATTTGATCAACGCATTCCAG-3’
PtrWRKY93	5’-TTCCAGGATCCATCAATCG-3’	5’-CGGAACTTGATACTGCTGGTG-3’
PtrWRKY95	5’-TTGTTGGGTCTGGAATTGAGG-3’	5’-ACACCGATAGTAGCTTCTGG-3’
PtoPR1.2	5'-TATAATAATCCCTCTATCCC-3’	5'-CTAGTAAGGACGCTGGCCAACG-3’
PtoPR2.3	5'-CAAAGGATTGCTTCCAGTCAAGC-3’	5'-TCAAGAAGGGCATCGAAGAGG-3’
PtoPR2.6	5'-ATGGGAGGTTATTCATCTAGAC-3’	5'-AATTGGCGGATGCTATTGCG-3’
PtoPR5.1	5'-ATGGCAACTTGCACGCATG-3’	5'-GACACCAGAGGCACAGTCAG-3’
PtoPR5.2	5'-CTGCTCCTTCTCTTCGTAAC-3'	5'-CTTTCCACTGCCTGATTCATC-3'
PtoPR5.3	5'-CACTTCTCTTGCTGCTTCTCC-3'	5'-CATTCAACTTGACCTGATCCG-3'
PtoPR5.4	5'-TTCCCCACTCTTATTTCCAGC-3'	5'- CCCATCTACAAGACTAACATCG-3'
PtoPR5.12	5'-TCTGGCCAGGAACTCTAACG-3'	5'-TTCTGCTAACGATGCTGGTGGG-3'
Pto18S	5’-GGCATGGAAGGTGATGCAGATC-3’	5’-CTGTGTCAAACAAGAACTTGTCC-3’
PtoSID2	5’-AGTTCAACACTTGTATGCAC-3’	5’-TTGATGATTCCTGAGTTCTC-3’
PtoMYB44.1	5’-GAGTTTATGTCGGTGATGC-3’	5’-GTTTCCTTTCTACCCTGTCC-3’
PtoMYB44.2	5’-CTGGCTCAAGTTCTGGATCC-3’	5’-TCGTTTAACCACAGCGTTCC-3’
PtoNPR1.1	5’-GTTGACCTAAATGAGACACC-3’	5’-TAATCTCAGCCTTGTCCTTG-3’
PtoAOS7	5'-CTATAACCAAGGCAGAGACGAG-3'	5'-GCTGAAGGCATGAAAGTACCTG-3'
PtoJAZ10	5’-AGAGCAGCAGCTGTTGAACTC-3’	5'-GAGGGAGAGCAGGAAATGAAGG-3'

Supplementary Table S3. Primers for quantitative real-time RT-PCR (qRT-PCR) and semi-qRT-PCR in this study. F and R represented the forward and reverse primers, respectively. Ptr and Pto meant *P. trichocarpa* and *P. tomentosa* Carr., respectively.

Supplemental Table S4. The number of various *cis*-acting elements in WRKY promoters

	SA	MeJA	Fungi		Wound		Cold	Salt/Drought		W-box
	TCA-element	CGTCA-motif	EIRE	ELI-box3	Box-S	WUN-motif	LTR	ABRE	MBS	
motif sequence	CCATCTTTTT	CGTCA	TTCGACC	AAACCAATT	AGCCACC	TCATTACGAA	CCGAAA	YACGTGGC	TAACTG	TTGACCY
WRKY1	1	0	0	0	0	0	0	0	0	0
WRKY2	3	0	0	0	0	0	0	0	1	0
WRKY3	1	0	0	0	0	1	0	0	0	0
WRKY4	1	1	0	1	0	0	0	0	1	2
WRKY5	0	1	0	0	0	0	0	0	1	3
WRKY6	1	3	1	0	0	0	0	0	2	0
WRKY7	1	4	0	0	0	1	0	0	1	4
WRKY8	1	0	0	0	0	0	0	0	1	0
WRKY9	1	0	0	0	0	1	1	0	1	1
WRKY10	1	2	0	0	0	0	1	0	1	1
WRKY11	1	0	0	0	0	0	0	0	0	2
WRKY12	0	1	0	0	0	0	0	0	1	0
WRKY13	0	0	0	0	0	0	1	0	0	1
WRKY14	1	0	0	0	0	0	0	0	0	1
WRKY15	0	0	0	0	0	0	5	0	0	2
WRKY16	1	0	0	0	0	0	2	0	1	2
WRKY17	0	0	0	0	0	1	0	0	0	1
WRKY18	0	0	0	0	0	0	0	0	2	5
WRKY19	0	2	0	0	0	0	1	0	0	1
WRKY20	0	0	0	0	0	0	0	0	0	0
WRKY21	1	1	0	0	0	0	2	0	2	1
WRKY22	1	0	0	0	0	0	1	0	0	0
WRKY23	0	2	1	0	1	1	0	0	0	3
WRKY24	0	0	0	0	0	0	1	0	0	2
WRKY25	0	1	0	0	0	0	0	0	0	1
WRKY26	1	1	0	0	0	0	0	0	0	0
WRKY27	0	1	0	0	1	0	0	0	0	0
WRKY28	2	0	0	0	0	1	0	0	0	1
WRKY29	5	2	0	0	0	0	0	0	1	0
WRKY30	0	1	1	0	0	0	0	0	0	0
WRKY31	2	0	0	0	0	1	0	0	1	1
WRKY32	0	3	0	0	0	0	0	1	0	2
WRKY33	1	0	0	0	0	0	0	1	1	1
WRKY34	0	2	0	0	0	0	0	1	2	3
WRKY35	1	1	0	0	0	0	1	1	0	0
WRKY36	2	1	0	0	0	0	1	1	4	1
WRKY37	1	0	0	0	0	0	0	0	0	0
WRKY38	3	1	0	0	0	0	0	1	1	0
WRKY39	3	0	0	0	1	0	1	2	0	1
WRKY40	2	0	0	0	0	0	0	1	0	0
WRKY41	2	0	0	0	0	0	0	1	2	0
WRKY42	2	4	0	0	0	0	0	3	2	1

WRKY43	1	1	0	0	0	0	0	0	3	1
WRKY44	0	1	0	0	0	0	0	2	0	1
WRKY45	1	0	0	0	0	0	1	0	2	0
WRKY46	3	1	0	0	1	0	1	4	4	0
WRKY47	0	0	0	0	0	0	0	0	0	0
WRKY48	0	1	0	0	0	0	0	1	3	0
WRKY49	0	3	0	0	0	0	0	0	1	1
WRKY50	2	3	0	0	0	0	3	0	5	2
WRKY51	0	4	0	0	0	1	0	6	4	1
WRKY52	3	2	0	0	0	0	0	2	3	0
WRKY53	0	1	0	0	0	0	1	1	3	0
WRKY54	0	0	0	1	0	0	1	0	1	2
WRKY55	2	0	0	0	0	0	1	1	0	0
WRKY56	1	1	0	0	0	0	0	1	2	1
WRKY57	1	3	1	0	0	0	0	1	4	1
WRKY58	3	0	0	0	1	1	3	4	2	2
WRKY59	1	2	0	0	0	0	0	0	1	2
WRKY60	4	1	0	0	1	0	1	3	3	7
WRKY61	1	1	0	0	0	1	2	2	1	4
WRKY62	1	3	0	1	0	0	1	1	1	2
WRKY63	0	2	0	0	0	0	0	2	1	0
WRKY64	2	2	0	0	1	0	0	1	2	2
WRKY65	1	3	0	1	0	0	5	1	2	1
WRKY66	0	3	0	0	0	0	0	0	1	0
WRKY67	1	1	2	0	2	0	0	2	5	2
WRKY68	0	1	0	0	0	1	1	0	3	1
WRKY69	2	1	0	0	0	0	0	1	1	0
WRKY70	2	1	0	0	0	0	0	0	4	1
WRKY71	3	1	0	0	0	0	0	4	2	2
WRKY72	2	1	0	0	0	0	0	0	3	3
WRKY73	1	2	0	0	0	0	1	2	3	2
WRKY74	2	0	0	1	2	0	1	3	2	3
WRKY75	2	0	0	1	0	1	1	2	1	1
WRKY76	2	0	0	0	0	0	0	0	4	2
WRKY77	0	2	0	0	0	0	0	1	2	11
WRKY78	3	6	0	0	0	0	0	3	4	3
WRKY79	3	1	0	0	0	0	1	2	0	1
WRKY80	0	2	0	1	1	0	0	0	2	2
WRKY81	3	0	0	0	0	0	1	3	1	3
WRKY82	0	2	1	0	1	1	1	3	3	3
WRKY83	3	4	0	0	0	0	0	1	2	1
WRKY84	0	3	0	0	0	0	0	2	2	1
WRKY85	1	3	1	0	0	0	0	1	3	5
WRKY86	1	1	0	1	0	0	0	5	2	0
WRKY87	3	1	2	0	1	0	2	2	1	1
WRKY88	4	3	0	0	0	0	0	5	0	1

WRKY89	0	4	0	0	0	0	1	0	3	2
WRKY90	0	1	0	1	0	0	0	0	0	1
WRKY91	3	2	0	0	0	0	0	0	2	2
WRKY92	2	4	0	0	0	0	2	1	0	0
WRKY93	3	1	0	0	0	0	1	0	2	0
WRKY94	0	0	0	0	0	0	1	1	1	0
WRKY95	0	0	0	0	0	0	0	1	0	3
WRKY96	3	0	0	0	0	0	0	0	1	1
WRKY97	3	0	0	0	0	0	0	1	0	0
WRKY98	0	1	0	0	0	0	0	1	2	0
WRKY99	2	2	0	0	0	0	1	1	0	1
WRKY100	0	0	0	0	0	0	0	0	1	3

Supplementary Table S4. Various *cis*-acting elements responsive to tresses in WRKY promoters.

The figures represented the number of each kind of elements.

Supplemental Table S5. Results of DGE analysis								
gene name	sequence ID VISION 3.0	sequence ID VISION 2.0	log2 Ratio (SA/WT)	log2 Ratio (MeJA/CK)	log2 Ratio (Mb/CK)	log2 Ratio (WOUND/CK)	log2 Ratio (COLD/CK)	log2 Ratio (SALT/CK)
PtrWRKY1	Potri.014G164300	POPTR_0014s16220.1	1.39	1.19	1.41	N/A	N/A	N/A
PtrWRKY2	Potri.011G169300	POPTR_0011s17230.1	-1.14	-0.22	-0.05	-1.05	-0.69	0.20
PtrWRKY3	Potri.008G091900	POPTR_0008s09140.1	2.07	1.08	2.37	1.71	-0.21	1.50
PtrWRKY4	Potri.017G088300	POPTR_0017s12430.1	0.14	0.47	0.39	0.29	-0.13	1.26
PtrWRKY5	Potri.002G221600	POPTR_0002s22180.1	1.86	2.00	2.08	0.62	1.45	2.91
PtrWRKY6	Potri.004G007500	POPTR_0004s00890.1	3.63	5.26	7.63	6.51	2.14	6.67
PtrWRKY7	Potri.002G123300	POPTR_0002s12480.1	1.37	0.43	0.72	0.77	0.27	2.56
PtrWRKY11	Potri.018G008500	POPTR_0018s03450.1	2.85	1.56	3.04	2.37	2.10	2.63
PtrWRKY15	Potri.005G141400	POPTR_0005s18490.1	1.98	1.50	1.28	1.21	0.23	2.62
PtrWRKY16	Potri.010G163000	POPTR_0010s17040.1	0.63	0.93	1.51	1.42	0.21	1.63
PtrWRKY17	Potri.006G072400	POPTR_0006s07170.1	0.64	0.40	1.46	0.10	-1.33	0.84
PtrWRKY18	Potri.006G263600	POPTR_0006s27950.1	7.78	5.77	9.58	6.83	4.59	6.42
PtrWRKY20	Potri.011G087900	POPTR_0011s09030.1	1.27	1.21	1.25	0.53	0.81	1.11
PtrWRKY21	Potri.005G219500	POPTR_0005s24100.1	0.96	0.12	1.35	-0.24	1.36	0.57
PtrWRKY22	Potri.002G164400	POPTR_0002s16590.1	-0.80	-0.35	1.22	2.79	1.04	0.95
PtrWRKY24	Potri.018G139300	POPTR_0018s13600.1	-0.29	-0.78	1.34	-0.26	-0.89	0.14
PtrWRKY28	Potri.002G059100	POPTR_0002s06000.1	0.31	1.61	1.82	-0.53	0.91	3.06
PtrWRKY30	Potri.012G031700	POPTR_0012s01380.1	10.55	11.50	12.14	9.97	9.40	11.90
PtrWRKY31	Potri.007G047400	POPTR_0007s10670.1	1.84	1.23	1.63	2.79	0.23	0.14
PtrWRKY32	Potri.018G107000	POPTR_0018s11630.1	1.37	0.84	0.62	0.57	0.81	0.85
PtrWRKY33	Potri.016G128300	POPTR_0016s13600.1	4.26	4.51	8.00	4.53	2.10	4.75
PtrWRKY35	Potri.018G019800	POPTR_0018s02470.1	5.81	3.33	5.74	12.06	8.62	14.08
PtrWRKY36	Potri.001G361600	POPTR_0001s37260.1	0.92	0.42	0.11	-0.30	0.41	1.20
PtrWRKY40	Potri.001G044500	POPTR_0001s03620.1	1.77	0.75	4.04	3.91	2.15	4.77
PtrWRKY41	Potri.001G092900	POPTR_0001s10490.1	5.78	5.41	7.58	2.53	1.23	4.72
PtrWRKY42	Potri.014G111900	POPTR_0014s10750.1	3.43	4.28	4.52	1.90	0.64	3.86
PtrWRKY47	Potri.002G228400	POPTR_0002s21330.1	1.37	1.93	4.05	6.18	4.44	1.95
PtrWRKY48	Potri.008G103300	POPTR_0008s10280.1	0.63	1.44	1.72	1.67	2.04	3.18
PtrWRKY49	Potri.006G087000	POPTR_0006s08730.1	2.60	1.03	1.46	-1.21	-0.77	1.05
PtrWRKY50	Potri.006G224100	POPTR_0006s24050.1	4.30	4.12	7.35	2.79	1.81	5.46
PtrWRKY51	Potri.005G085200	POPTR_0005s08720.1	16.28	12.70	16.21	12.70	9.26	14.63
PtrWRKY52	Potri.014G119800	POPTR_0014s11490.1	2.14	0.78	1.51	3.93	2.89	1.18
PtrWRKY53	Potri.003G138600	POPTR_0003s13840.1	4.80	4.73	9.19	5.69	3.64	4.08
PtrWRKY54	Potri.013G090300	POPTR_0013s09470.1	4.91	2.12	5.72	2.79	1.81	5.63
PtrWRKY59	Potri.002G195300	POPTR_0002s19630.1	1.59	1.31	1.19	0.97	1.35	1.22
PtrWRKY60	Potri.018G019700	POPTR_0018s02480.1	15.15	8.49	12.97	9.83	8.86	14.32
PtrWRKY61	Potri.015G064100	POPTR_0015s07530.1	9.36	10.75	12.26	9.36	N/A	9.29
PtrWRKY62	Potri.016G137900	POPTR_0016s14490.1	5.29	3.72	5.32	1.92	1.04	3.50
PtrWRKY63	Potri.002G168700	POPTR_0002s17010.1	2.40	2.58	6.22	3.08	1.60	2.53
PtrWRKY64	Potri.014G096200	POPTR_0014s09190.1	2.51	2.43	6.34	1.69	0.28	1.95
PtrWRKY65	Potri.011G070100	POPTR_0011s06990.1	3.26	2.27	2.14	0.13	-0.94	-0.71
PtrWRKY69	Potri.004G060900	POPTR_0004s05920.1	2.22	2.86	3.82	1.08	-0.36	0.55
PtrWRKY70	Potri.019G123500	POPTR_0019s14460.1	3.57	2.42	3.96	3.38	2.14	5.18
PtrWRKY71	Potri.005G203200	POPTR_0005s22480.1	-2.17	0.31	1.24	1.79	1.71	1.62

PtrWRKY73	Potri.013G153400	POPTR_0013s14960.1	3.51	2.84	5.29	3.75	1.97	5.17
PtrWRKY74	Potri.006G105300	POPTR_0006s10600.1	1.46	2.67	4.25	1.62	0.45	1.01
PtrWRKY75	Potri.T043800	POPTR_0017s09750.1	12.13	10.61	12.51	N/A	N/A	10.31
PtrWRKY76	Potri.013G042600	POPTR_0013s03990.1	1.05	1.36	1.20	-0.25	0.47	2.26
PtrWRKY77	Potri.003G182200	POPTR_0003s18060.1	1.37	0.99	3.72	5.86	3.85	3.36
PtrWRKY78	Potri.011G007800	POPTR_0021s00280.1	4.05	5.17	7.23	7.45	4.23	6.24
PtrWRKY80	Potri.015G099200	POPTR_0015s11130.1	10.02	N/A	11.43	N/A	N/A	13.18
PtrWRKY83	Potri.014G155100	POPTR_0014s15320.1	2.43	1.12	1.58	3.95	1.38	1.26
PtrWRKY84	Potri.002G186600	POPTR_0002s18770.1	1.63	4.51	4.36	2.08	0.23	2.55
PtrWRKY85	Potri.010G147700	POPTR_0010s15750.1	0.92	2.34	3.72	3.24	2.45	2.76
PtrWRKY86	Potri.002G193000	POPTR_0002s19390.1	2.70	1.71	1.33	3.01	1.23	5.23
PtrWRKY87	Potri.016G099900	POPTR_0016s10610.1	-3.65	-11.88	-11.88	-3.49	0.99	-1.24
PtrWRKY88	Potri.007G079800	POPTR_0007s06720.1	13.97	10.00	13.54	9.34	N/A	12.28
PtrWRKY89	Potri.006G109100	POPTR_0006s10950.1	8.60	4.19	6.31	3.96	2.60	4.64
PtrWRKY91	Potri.008G094000	POPTR_0008s09360.1	1.46	2.62	3.31	2.62	0.23	1.14
PtrWRKY93	Potri.001G352400	POPTR_0001s34520.1	2.86	4.93	5.50	2.21	1.23	7.08
PtrWRKY95	Potri.003G169100	POPTR_0003s16750.1	13.79	13.92	14.24	13.53	9.64	16.99

Supplementary Table S5. Results of DGE analysis. The common names and gene IDs of PtrWRKY genes from different visions of Phytozome. The log2 expression values of salicylic acid (SA), Methyl Jasmonate (MeJA), *Marssonina brunnea* f.sp. *multigermtubi* (Mb) inoculation and wounding, cold, salinity treatments compared with CK were listed. N/A represented that mRNA transcripts of WRKY genes were not detected. Minus represented down-regulation of the gene.
